# Stem cell-derived exosomes in the therapy of renal diseases: a comprehensive review

**DOI:** 10.1186/s13287-026-05199-8

**Published:** 2026-08-01

**Authors:** Qikai Luo, Nan Yang, Yiwei Shang, Yixuan Gao, Qiang He, Juan Jin

**Affiliations:** 1 Department of Nephrology, The First Affiliated Hospital of Zhejiang Chinese Medical University (Zhejiang Provincial Hospital of Chinese Medicine), Hangzhou, 310006 Zhejiang People’s Republic of China; 2 Zhejiang Key Laboratory of Research and Translation for Kidney Deficiency-Stasis-Turbidity Disease, Zhejiang-Macau International Joint Laboratory of Integrated Traditional Chinese and Western Medicine for Nephrology and Immunology, Zhejiang Chinese Medical University Integrated Research and Clinical Laboratory for Precision Prevention and Treatment of Metabolic and Kidney Diseases, Hangzhou, 310006 Zhejiang People’s Republic of China; 3 First Clinical Medical College, Wenzhou Medical University, Wenzhou, 325035 Zhejiang People’s Republic of China

## Abstract

Chronic kidney disease (CKD) represents a growing global public health challenge. Current treatments, including pharmacological therapy, immunosuppression, and renal replacement therapy, mainly aim to slow disease progression rather than restore damaged renal tissue, and their efficacy remains limited in many patients. Although stem cell-based therapies have shown regenerative potential, their clinical application is constrained by poor cell survival, limited engraftment, immunogenicity, and potential safety concerns. Stem cell-derived exosomes (SC-exosomes) have therefore attracted increasing attention as a potential cell-free therapeutic strategy. These nanoscale extracellular vesicles can transfer bioactive cargoes, including proteins, lipids, messenger RNAs (mRNAs), and microRNAs (miRNAs), to recipient cells and thereby mediate intercellular communication. Compared with parental stem cell transplantation, SC-exosomes may offer several advantages, including lower immunogenicity, reduced tumorigenic risk, relative stability, and the potential to cross certain biological barriers. This comprehensive review summarizes current research on SC-exosomes in renal diseases, with emphasis on their biological characteristics, therapeutic mechanisms, preclinical evidence, and translational challenges. SC-exosomes may contribute to renal repair by promoting cell survival and regeneration, suppressing inflammation, apoptosis, oxidative stress, and fibrosis, and modulating immune and microvascular responses. We also discuss their potential relevance in multiple renal conditions, including acute kidney injury (AKI), CKD, diabetic nephropathy (DN), and other kidney diseases, as well as the use of exosomal cargoes as candidate diagnostic or prognostic biomarkers. Finally, we outline emerging opportunities and unresolved challenges in this field, including engineered exosomes, targeted delivery, exosome heterogeneity, dose optimization, large-scale production, purification, quality control, and long-term safety. Further standardized preclinical studies and well-designed clinical trials are needed before SC-exosome-based approaches can be translated into routine clinical application.

## Introduction

Kidney disease is a major global health burden, and the increasing prevalence of chronic kidney disease (CKD) and end-stage kidney disease (ESKD) has imposed substantial clinical, social, and economic challenges [1]. Current therapeutic strategies, including pharmacological treatment, immunosuppression, and renal replacement therapy, can slow disease progression in many patients but have limited capacity to promote structural repair or functional recovery of injured renal tissue [2]. Therefore, regenerative and cell-free therapeutic strategies have attracted increasing attention in nephrology.

Stem cell-based therapy has been investigated as a potential regenerative approach for kidney diseases because of its immunomodulatory and tissue-repairing properties [3–5]. However, its clinical translation remains limited by several concerns, including low cell survival after transplantation, poor engraftment efficiency, possible immunogenicity, and potential tumorigenic risks [6–8]. Increasing evidence suggests that many of the beneficial effects of stem cells are mediated through paracrine mechanisms rather than direct cell replacement [8, 9]. In this context, stem cell-derived exosomes (SC-exosomes), a subset of small extracellular vesicles (EVs) approximately 40–160 nm in diameter, have emerged as promising cell-free therapeutic candidates [8, 10, 11].

SC-exosomes carry bioactive molecules derived from their parental cells, including proteins, lipids, messenger RNAs (mRNAs), microRNAs (miRNAs), and other non-coding RNAs [10, 12]. Through intercellular transfer of these cargoes, SC-exosomes may modulate key pathological processes involved in kidney injury, such as inflammation, oxidative stress, apoptosis, epithelial–mesenchymal transition (EMT), fibrosis, immune dysregulation, and microvascular injury [13–15]. Compared with direct stem cell transplantation, SC-exosomes may offer several practical advantages, including lower cellular safety concerns, easier storage and handling, reduced immunogenicity, and the potential to be engineered as drug delivery vehicles [8, 9, 16]. Nevertheless, most current evidence is derived from in vitro experiments and animal models, and the clinical efficacy and safety of SC-exosomes in kidney diseases require further validation [17, 18].

This comprehensive review summarizes the biogenesis, isolation, characterization, and biological functions of SC-exosomes, with a particular focus on their therapeutic mechanisms in renal diseases. We discuss available evidence in acute kidney injury, renal ischemia–reperfusion injury, chronic kidney disease, diabetic nephropathy, IgA nephropathy, lupus nephritis, renal cancer, and other renal disorders. In addition, we examine the roles of exosomes as candidate biomarkers and drug delivery systems, while critically discussing major translational challenges, including exosome heterogeneity, dose optimization, delivery efficiency, large-scale production, quality control, and long-term safety [16–19].

## Key points

Chronic kidney disease (CKD) is a growing global health burden, and current pharmacological, immunosuppressive, and renal replacement therapies mainly slow disease progression rather than restore damaged renal tissue.

Stem cell-based therapies have shown regenerative and immunomodulatory potential, but their clinical translation remains limited by poor cell survival, low engraftment efficiency, possible immunogenicity, and safety concerns.

Stem cell-derived exosomes (SC-exosomes) are nanoscale lipid-bilayer extracellular vesicles that can transfer bioactive cargoes from parental stem cells and may retain part of their paracrine and immunomodulatory activity, with fewer cell-related safety concerns than direct stem cell transplantation.

Preclinical studies have investigated SC-exosomes in acute kidney injury, chronic kidney disease, diabetic nephropathy, renal fibrosis, and less-studied renal diseases such as polycystic kidney disease; however, their disease-specific efficacy and clinical applicability remain to be validated.

SC-exosomes may contribute to renal repair by mediating intercellular communication, transferring bioactive cargoes, modulating inflammatory and fibrotic signaling, and serving as potential delivery vehicles for therapeutic molecules.

Engineered and production-optimized SC-exosomes may improve cargo loading, tissue targeting, and manufacturing consistency, but standardization, dose optimization, delivery efficiency, and long-term safety remain major translational challenges.

## Stem cell and exosome biogenesis

**Fig. 1:**
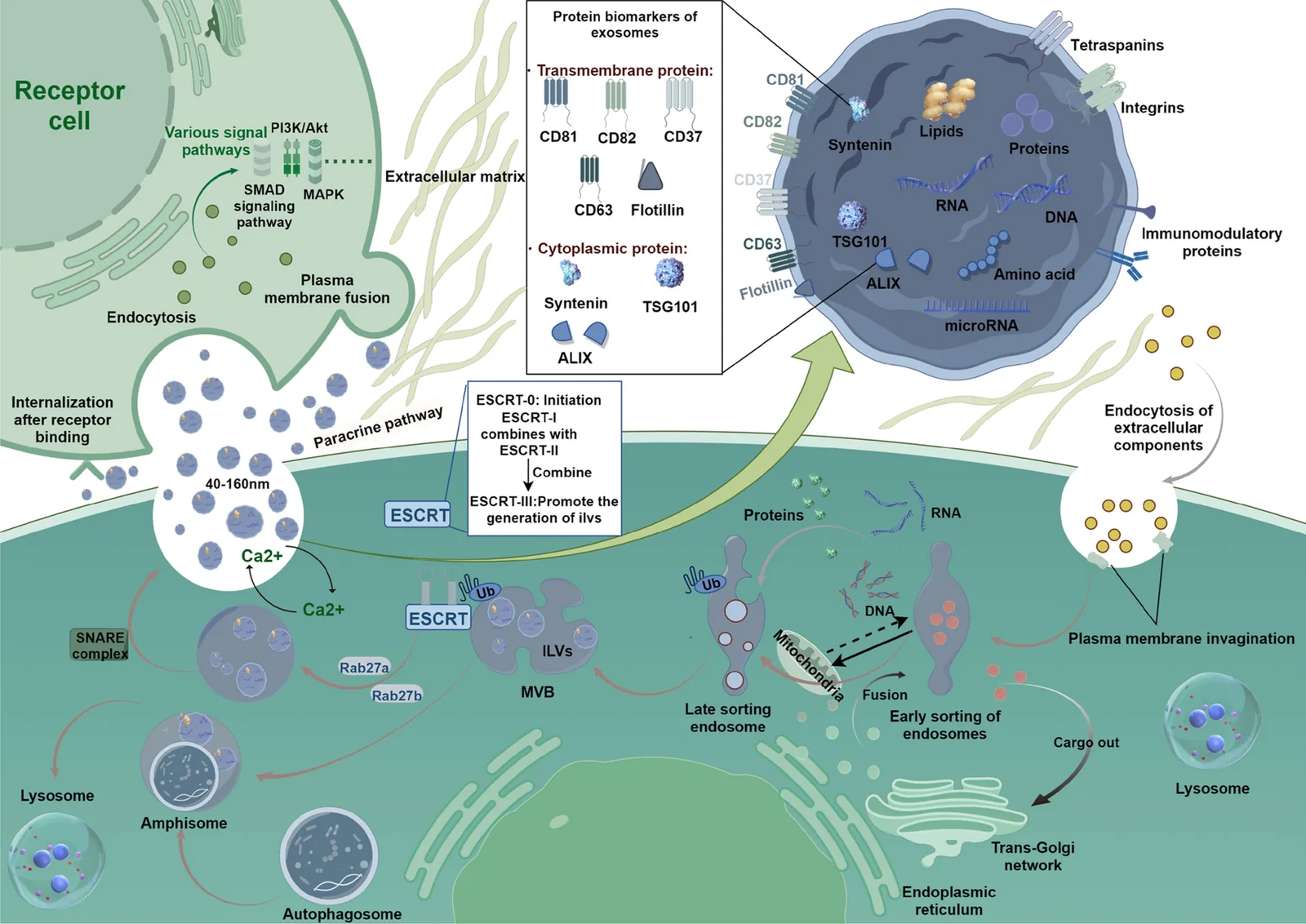
Biogenesis, secretion, cargo composition, and cellular uptake of exosomes. Exosomes are generated through the endosomal pathway. Plasma membrane invagination gives rise to early-sorting endosomes, which mature into late-sorting endosomes and multivesicular bodies (MVBs) containing intraluminal vesicles (ILVs). During ILV formation, cytoplasmic proteins, lipids, nucleic acids, and other bioactive molecules can be incorporated into vesicles through cargo-sorting processes, including ESCRT-associated mechanisms. MVBs may either fuse with lysosomes or autophagic compartments for degradation, or traffic to the plasma membrane, where Rab27A/Rab27B and SNARE-associated membrane fusion facilitate the release of ILVs as exosomes. Released exosomes contain characteristic proteins such as TSG101, ALIX, CD63, CD81, and CD82, as well as lipids, proteins, nucleic acids, and other bioactive molecules. They can be taken up by recipient cells through membrane fusion, endocytosis, or receptor-mediated internalization, thereby modulating downstream signaling pathways such as TGF-β, MAPK, and PI3K/Akt signaling

Exosomes are small lipid bilayer extracellular vesicles (EVs) that are widely present in body fluids such as blood, urine, and saliva. They are approximately 40–160 nm in diameter, with an average diameter of about 100 nm, and carry diverse cellular components, including nucleic acids, proteins, lipids, amino acids, and metabolites [10]. Exosomes were first observed by Trams et al. in cell culture using electron microscopy in 1981 [20]. In 1987, Johnstone and colleagues further characterized vesicles released from sheep reticulocytes and formally named them exosomes [21]. Exosome biogenesis is a complex process that is mainly mediated through the endosomal sorting complex required for transport (ESCRT) pathway [22]. During this process, extracellular materials and plasma membrane proteins are internalized through endocytosis and plasma membrane invagination to form early-sorting endosomes (ESEs). ESEs can exchange material with other organelles or fuse with one another to generate late-sorting endosomes (LSEs). LSEs subsequently mature into multivesicular bodies (MVBs) through inward membrane budding, forming intraluminal vesicles (ILVs) that incorporate cytoplasmic constituents. MVBs may either fuse with autophagosomes or lysosomes for degradation or migrate to the cell periphery under the guidance of Rab27A and Rab27B. Fusion of MVBs with the plasma membrane is facilitated by the soluble *N*-ethylmaleimide-sensitive factor attachment protein receptor (SNARE) complex, leading to extracellular release of ILVs as exosomes [10, 23, 24]. Ca^2+^ has also been proposed to facilitate exosome release [25]. Released exosomes can enter recipient cells through plasma membrane fusion, receptor-mediated internalization, or endocytosis [26]. Exosomes contain a diverse array of proteins, lipids, DNA, and a wide range of RNAs [12], which play a variety of roles (e.g., signaling, immune response, inflammatory response) by transporting these substances into the recipient cell or by targeting the binding of particular receptors on the cell membrane, thereby inhibiting or activating specific signaling pathways [22]. For example, exosomes can modulate Smad, phosphatidylinositol 3-kinase (PI3K)/protein kinase B (Akt), and other signaling pathways involved in cell proliferation, differentiation, and apoptosis [27, 28]. The International Society for Extracellular Vesicles (ISEV) has identified cytoplasmic proteins (syntenin, TSG101, and ALIX), transmembrane proteins (e.g., CD81, CD82, CD37, and CD63), and negative marker proteins (e.g., albumin) as useful markers for exosome characterization [11]. Their nanoscale size, diverse cargo composition, and characteristic markers support the central role of exosomes in intercellular communication. Understanding exosome biogenesis and secretion is therefore important for studying their therapeutic relevance in kidney disease (Fig. 1).

Stem cells can self-renew and, under appropriate conditions, differentiate into diverse functional cell types involved in tissue regeneration and disease treatment. Mesenchymal stem cells (MSCs) are commonly used in cell therapy research because of their relative ease of isolation and culture [3]. MSCs can home to injured tissues and contribute to tissue repair in kidney disease [4, 5], but their survival and engraftment after injection are limited, and long-term MSC implantation is uncommon. In addition, MSCs share some gene-expression features with mesenchymal tumor cells, raising concerns about potential tumorigenic risk [6]. MSCs may also contribute to tumor growth through mechanisms such as promoting angiogenesis [7]. These limitations indicate that MSC therapy does not always produce the desired therapeutic outcome and underscore the need for safer and more effective alternatives. Exosomes, a specific subtype of extracellular vesicles (EVs), have been implicated in tissue repair and inflammatory diseases [29]. For example, hucMSC-exosomal miR-874-3p has been reported to attenuate renal tubular epithelial cell injury by targeting the RIPK1/PGAM5 axis [30]. Compared with stem cells, nanosized exosomes may cross certain biological barriers more readily and have advantages such as low immunogenicity, ease of isolation and storage, lack of infusion toxicity, and fewer ethical concerns [8]. At the same time, exosomes may also participate in disease pathophysiology, particularly in renal diseases [13]. Clarifying their disease-related mechanisms may help guide therapeutic development and address some limitations of conventional therapies and direct stem cell transplantation.

Recent studies have shown that stem cell-derived exosomes (SC-exosomes) originate from various stem cell sources. MSC-exosomes are the most widely studied and may contribute to the treatment of chronic kidney disease (CKD) and other disorders by retaining part of the biological activity of MSCs and serving as drug carriers. Human embryonic stem cell (hESC)-derived exosomes may support tissue regeneration and repair, including cardiac repair, whereas induced pluripotent stem cell (iPSC)-derived exosomes may exert angiogenic, pro-proliferative, and anti-apoptotic effects in multiple disease models. Exosomes derived from hematopoietic and other stem cell types have also been reported [14, 31, 32]. SC-exosomes not only retain some properties of their parental stem cells but also provide distinct advantages. They contain bioactive molecules such as transforming growth factor-β (TGF-β), vascular endothelial growth factor (VEGF), interleukin-6 (IL-6), chemokines, immunomodulatory factors, and growth factors [15], which may contribute to immunomodulation, cell growth, and tissue repair. Increasing evidence suggests that many effects of stem cell therapy are mediated by paracrine mechanisms, with exosomes representing an important functional component [33]. Thus, SC-exosomes may retain part of the paracrine activity of their parental stem cells, including anti-inflammatory, immunomodulatory, and tissue-repair-associated effects, while reducing concerns related to live-cell transplantation, such as poor engraftment, uncontrolled cell persistence, and cell-associated tumorigenic risk [9, 34]. Compared with direct stem cell therapy, SC-exosomes may serve as mediators of intercellular communication, carriers for biological or chemical drugs, candidate biomarkers for kidney disease, and relatively stable vesicles that are easier to isolate and store [10, 35, 36]. These properties support the investigation of SC-exosomes as cell-free therapeutic candidates for renal diseases, although their efficacy and safety remain to be validated in standardized preclinical and clinical studies. Their nanoscale size may allow them to penetrate dense tissues, carry DNA, small interfering RNAs (siRNAs), miRNAs, and proteins, mediate long-distance intercellular communication, and regulate specific signaling pathways [29]. Currently, SC-exosomes have been investigated in multiple disease models, including kidney injury, endocrine disorders, gastrointestinal disorders, cardiovascular diseases, lung diseases, cancer, orthopedic conditions, and neurodegenerative diseases [9, 16, 37].

## Generation, capture, isolation, and preservation of exosomes

Exosome production and biological activity can be enhanced through several approaches, including biochemical stimuli [e.g., inflammatory mediators, bone morphogenetic protein-2 (BMP-2), and lithium ions], physical stimuli, and environmental stressors such as hypoxia or nutrient deprivation [38]. Elevated Ca^2+^ levels may further facilitate exosome formation, and the combined effects of these factors may increase both exosome yield and biological activity [39]. Accurate characterization is essential to ensure the quality and function of active exosome preparations. Exosomes are commonly characterized according to morphology, marker expression, and size using techniques such as nanoparticle tracking analysis (NTA), cryo-electron microscopy (cryo-EM), Raman tweezers microspectroscopy (RTM), confocal microscopy, electron microscopy, and atomic force microscopy (AFM). Cryo-EM avoids artifacts caused by chemical treatment and dehydration, whereas AFM allows nanometer-scale visualization with high resolution, supporting accurate exosome characterization [40, 41]. Emerging techniques, including electrochemical biosensing systems, surface plasmon resonance (SPR), quartz crystal microbalance (QCM), and fluorescence-based single-vesicle analysis, may further support exosome analysis in clinical and translational applications [42].

Various methods can be used to isolate exosomes according to their physical and biochemical properties, including particle size, density, surface markers, and charge characteristics [16]. Commonly used isolation strategies include differential ultracentrifugation, density gradient centrifugation, ultrafiltration (UF), size-exclusion chromatography (SEC), immunoaffinity capture, polymer-based precipitation (PBP), microfluidic isolation, and hybrid or combined approaches. Because no single method can simultaneously achieve optimal yield, purity, processing efficiency, scalability, and preservation of biological activity, the selection of an isolation strategy should be determined according to the sample type, experimental purpose, and downstream application. Therefore, these methods are compared in Table 1 in terms of principle, yield, purity, processing time, scalability, advantages, specific limitations, and suitable applications.

**Table 1:** Comparison of exosome isolation mechanisms

Isolation method	Principle	Yield	Purity	Processing time	Scalability	Advantage	Specific limitations	Suitable applications	References
Differential ultracentrifugation	Sequential centrifugation based on particle size and density	Moderate to high	Moderate	Long	Moderate	Widely used; suitable for processing relatively large sample volumes; does not require specific binding reagents	Time-consuming; requires expensive equipment; may cause vesicle aggregation or deformation; co-isolation of protein aggregates, lipoproteins, and other extracellular vesicles may occur	Basic research; mechanistic studies; preparation of exosomes from cell culture supernatants	[44–46]
Density gradient centrifugation	Separation according to buoyant density using sucrose or iodixanol gradients	Low to moderate	High	Long	Low to moderate	Provides higher purity than conventional ultracentrifugation; useful for reducing contamination by proteins and non-vesicular particles	Labor-intensive; relatively low recovery; requires technical expertise; not ideal for high-throughput or large-scale production	High-purity exosome preparation; proteomic and RNA profiling; validation studies	[42, 44, 46]
Ultrafiltration	Size-based enrichment using membranes with defined molecular-weight cutoffs	Moderate to high	Low to moderate	Short to moderate	Moderate to high	Simple, rapid, and cost-effective; suitable for concentrating large sample volumes; does not require ultracentrifugation	Membrane clogging may reduce yield; shear stress may damage vesicles; soluble proteins and similarly sized particles may be retained	Sample concentration; preliminary enrichment before downstream purification; large-volume biological fluids	[16, 44, 47]
Size-exclusion chromatography	Separation based on hydrodynamic size through porous beads	Moderate	Moderate to high	Moderate	Moderate	Preserves vesicle integrity; relatively reproducible; reduces soluble protein contamination; compatible with functional studies	Sample dilution may occur; limited loading volume; possible co-isolation of similarly sized particles such as lipoproteins	Functional assays; biomarker studies; preparation requiring preserved vesicle activity	[42, 46, 48]
Polymer-based precipitation	Reduction of exosome solubility using polymers such as polyethylene glycol	High	Low	Short	High	Easy to perform; does not require specialized equipment; suitable for parallel processing of multiple samples	Co-precipitation of abundant proteins, polymer residues, lipoproteins, and non-exosomal particles; lower purity may affect downstream molecular analyses	Initial screening; exploratory biomarker discovery; situations requiring high recovery rather than high purity	[42, 44, 46]
Immunoaffinity capture	Antibody- or ligand-based capture of exosomes expressing specific surface markers such as CD9, CD63, or CD81	Low to moderate	High	Moderate	Low	High specificity; enables enrichment of defined exosome subpopulations; useful for disease-specific marker detection	Expensive; limited by antibody specificity and marker heterogeneity; may miss exosomes lacking the selected marker; difficult to scale up	Targeted biomarker detection; isolation of specific exosome subpopulations; clinical diagnostic assays	[42, 46]
Microfluidic isolation	Integration of size-, immunoaffinity-, acoustic-, or electric-field-based separation on microchips	Low to moderate	Moderate to high	Short	Low at present	Requires small sample volumes; rapid and potentially automated; suitable for point-of-care or diagnostic platforms	Device fabrication and standardization remain challenging; limited throughput; clinical-scale production is not yet established	Liquid biopsy; diagnostic testing; small-volume clinical samples; rapid exosome analysis	[42, 46, 49]
Hybrid or combined isolation strategies	Sequential or integrated use of two or more methods, such as ultrafiltration plus SEC, precipitation plus SEC, or tangential-flow filtration plus chromatography	Moderate to high	Moderate to high	Moderate to long	Moderate to high, depending on the platform	Balances yield and purity; reduces method-specific contaminants; may improve reproducibility and suitability for translational studies	Requires protocol optimization; increased cost and operational complexity; lack of universal standardization across sample types	Translational research; large-scale therapeutic production; preparation of clinical-grade exosome products	[16–19, 42]

Commercial isolation kits can provide rapid and convenient enrichment of exosomes; however, their broader application remains limited by exosome heterogeneity, co-isolation of non-exosomal contaminants, batch-to-batch variability, and insufficient standardization [42]. Recent studies have also explored alternative or combined strategies to improve recovery and purity. For example, titanium dioxide (TiO₂) can specifically and reversibly bind to phosphate groups in the exosomal lipid bilayer, providing a potential approach for efficient exosome isolation [43]. Although ultracentrifugation remains one of the most widely used conventional methods for exosome separation, its limitations, including time consumption, equipment requirements, vesicle aggregation, and possible co-isolation of contaminants, should be considered when selecting isolation protocols [9, 44].

## Stem cell-derived exosomes for kidney diseases

### Functions and therapeutic mechanisms of SC-exosomes

The molecular and cellular diversity of exosomes underlies their broad relevance in biomedical research and renal disease studies. Exosomes are released by many cell types, including immune cells, epithelial cells, endothelial cells, renal tubular epithelial cells, podocytes, and mesenchymal cells, and they can be detected in body fluids such as blood, urine, and saliva. By transferring proteins, lipids, mRNAs, miRNAs, and other non-coding RNAs to recipient cells, exosomes participate in intercellular communication and may influence both physiological and pathological signaling [10, 12].

In kidney diseases, the biological effects of exosomes depend on their cellular origin, cargo composition, and disease context. Disease-derived exosomes, such as those released from injured renal tubular epithelial cells, podocytes, immune cells, cystic epithelial cells, or tumor cells, may contribute to inflammation, fibroblast activation, podocyte dysfunction, cyst progression, or tumor-related processes [13, 49–51]. In contrast, SC-exosomes, particularly those derived from mesenchymal stem cells, are mainly investigated as cell-free therapeutic candidates because they can transfer bioactive cargoes from parental stem cells and may modulate renal injury, inflammation, fibrosis, and repair responses [14, 17].

The reported protective effects of SC-exosomes in renal disease models involve several overlapping but context-dependent mechanisms. In inflammatory kidney injury and diabetic kidney disease, SC-exosomes have been reported to suppress inflammatory activation, regulate macrophage polarization, and modulate inflammasome-related signaling through pathways involving miR-146b, miR-146a-5p, NLRP3, and indoleamine 2,3-dioxygenase (IDO)-associated immunoregulation [52–55]. In AKI and renal ischemia–reperfusion injury models, SC-exosomes may reduce tubular epithelial apoptosis, improve mitochondrial function, promote tubular repair, attenuate ferroptosis, and enhance antioxidant responses through mechanisms involving miR-125b-5p/p53, Sox9 activation, lncRNA TUG1, and Nrf2-related signaling [56–61]. In chronic or diabetic kidney injury models, SC-exosomes may attenuate epithelial–mesenchymal transition, extracellular matrix deposition, and renal fibrosis through let-7c-, miR-21a-5p-, miR-374a-5p-, anti-let-7i-5p-, Hedgehog/SMO-, and miR-424-5p-related pathways [62–67]. In addition, MSC-derived EVs have been reported to improve renal microvascular injury in metabolic renovascular disease [68]. These findings suggest that the renal effects of SC-exosomes are mediated by disease-specific cargo networks, including miRNAs, long non-coding RNAs (lncRNAs), circular RNAs (circRNAs), and proteins, rather than by a single universal pathway [55, 61, 69].

Several pathways recur across different renal disease models. Inflammation-related effects are commonly associated with NF-κB, NLRP3, TLR4, TRAF6/STAT1, and macrophage-polarization pathways. Anti-apoptotic and pro-reparative effects are frequently linked to PTEN/Akt, PI3K/Akt, p53-related signaling, and tubular epithelial regeneration pathways. Antioxidant effects are often associated with Nrf2/Keap1 or Nrf2/ARE signaling, whereas anti-fibrotic effects are mainly related to TGF-β-associated signaling, Smad-dependent pathways, mTOR, Hedgehog/SMO, MAPK6/MK5/YAP, glycolytic regulation, and extracellular matrix remodeling. Accumulating evidence has identified the critical role of miRNAs in kidney diseases and inflammatory diseases [70]. Representative SC-exosomal miRNA cargoes, their renal disease contexts, target pathways, biological effects, and evidence levels are summarized in Table 2.

**Table 2:** Representative miRNA cargoes in stem cell-derived exosomes or EVs and their mechanistic relevance in renal diseases

miRNA cargo	Exosome/EV source	Renal disease context	Experimental model/evidence type	Target/pathway and mechanistic direction	Major biological effect	Evidence level/relative priority	References
miR-125a	Adipose-derived mesenchymal stem cell exosomes (adMSC-exosomes)	Diabetic nephropathy; renal fibrosis	Mesangial cell-related experimental models	Targets HDAC1 and downregulates ET-1 expression in mesangial cells	Reduces mesangial cell injury and attenuates renal fibrotic responses	Preclinical evidence; moderate priority because of a defined anti-fibrotic pathway	[79]
miR-486	Adipose-derived stem cell exosomes (ADSC-exosomes)	Diabetic nephropathy; podocyte injury	Podocyte injury models	Inhibits the Smad1/mTOR pathway and restores podocyte autophagy	Promotes autophagy flux and reduces podocyte apoptosis	Preclinical evidence; high priority for podocyte protection in diabetic nephropathy	[80]
miR-26a-5p	Adipose-derived mesenchymal stem cell extracellular vesicles	Diabetic nephropathy; podocyte dysfunction	Diabetic nephropathy-related in vitro and in vivo models	Targets TLR4 and suppresses VEGFA/NF-κB-mediated inflammatory signaling	Reduces inflammatory activation and podocyte injury	Preclinical evidence; high priority because of disease-specific validation and a defined inflammatory pathway	[81]
miR-215-5p	ADSC-exosomes	Diabetic nephropathy; epithelial–mesenchymal transition (EMT)	Podocyte EMT-related experimental models	Targets ZEB2 and inhibits EMT-associated transcriptional activation	Attenuates podocyte EMT and protects the podocyte phenotype	Preclinical evidence; moderate priority for EMT-related podocyte protection	[82]
miR-16-5p	Human urine-derived stem cell exosomes (hUSC-exosomes)	Diabetic nephropathy; podocyte injury	Podocyte injury models and diabetic nephropathy-related experimental evidence	Targets VEGFA and reduces VEGFA-associated podocyte injury	Promotes podocyte survival and reduces podocyte apoptosis	Preclinical evidence; moderate priority because of podocyte-specific relevance	[83, 84]
let-7c	Bone marrow mesenchymal stem cell exosomes (BMSC-exosomes)	Renal fibrosis; chronic kidney disease	Renal fibrosis models	Suppresses TGF-β1-driven pro-fibrotic signaling	Protects renal tubular epithelial cells and attenuates renal fibrosis	Preclinical evidence; high priority for anti-fibrotic mechanisms	[62]
miR-125b	BMSC-exosomes	Diabetic nephropathy; tubular injury	Diabetic nephropathy-related experimental models	Regulates the TRAF6/Akt axis and attenuates inflammatory injury signaling	Protects renal tubular cells and suppresses disease progression	Preclinical evidence; moderate priority because of defined inflammatory and survival-related signaling	[85]
miR-222	BMSC-derived extracellular vesicles	Diabetic nephropathy; mesangial matrix accumulation	Hyperglycaemic mesangial cell models	Modulates STAT5 and reduces TGF-β-associated extracellular matrix production	Decreases mesangial collagen production and matrix accumulation	In vitro/preclinical evidence; moderate priority for mesangial cell injury	[86]
miR-21a-5p	BMSC-exosomes	Renal fibrosis; tubular epithelial metabolic reprogramming	Renal fibrosis-related experimental models	Targets PFKM and suppresses glycolysis-associated renal fibrotic activation	Preserves tubular epithelial cells and reduces renal fibrosis	Preclinical evidence; high priority because of a defined metabolic anti-fibrotic mechanism	[63]
miR-199a-3p	BMSC-exosomes	Renal ischemia–reperfusion injury (RIRI)	Ischemia–reperfusion injury models	Downregulates Sema3A and activates Akt/ERK-mediated tubular cell survival signaling	Reduces organelle stress and tubular cell apoptosis	Preclinical evidence; high priority for RIRI-associated tubular protection	[87]
miR-381-3p	BMSC-exosomes	Chronic kidney disease; vascular calcification	CKD-associated vascular calcification models	Targets NFAT5 and suppresses calcification- and apoptosis-related signaling	Reduces vascular calcification and apoptosis	Preclinical evidence; moderate priority for CKD-associated vascular complications	[88]
miR-146b	Human umbilical cord mesenchymal stem cell exosomes (hucMSC-exosomes)	Sepsis-associated acute kidney injury	Sepsis-associated AKI models	Suppresses IRAK1/NF-κB inflammatory signaling through miR-146b upregulation	Reduces renal inflammation, alleviates tubular injury, and improves renal outcomes in experimental AKI	Preclinical evidence; high priority for inflammation-driven AKI because of a clearly defined miRNA-target-pathway axis	[52, 89]
miR-874-3p	hucMSC-exosomes	Tubular epithelial injury; AKI-related renal damage	Tubular epithelial cell injury models	Regulates the RIPK1/PGAM5 axis and attenuates tubular epithelial injury signaling	Reduces tubular epithelial cell damage and promotes repair	Preclinical evidence; moderate priority because of a defined tubular injury pathway	[30]
miR-146a-5p	hucMSC-exosomes	Diabetic nephropathy; renal inflammation	Diabetic nephropathy models	Inhibits the TRAF6/STAT1 pathway and promotes M2 macrophage polarization	Reduces inflammation and improves renal function	Preclinical evidence; high priority for immunomodulation in diabetic nephropathy	[53]
miR-335-5p	hucMSC-exosomes	Renal tubular inflammation and tubular epithelial-myofibroblast transition	Renal tubular epithelial cell models	Targets ADAM19 and suppresses tubular inflammation and epithelial–myofibroblast transition	Reduces tubular inflammatory injury and inhibits pro-fibrotic cellular transition	Preclinical evidence; moderate priority for anti-inflammatory and anti-fibrotic effects	[90]

The relative priority was assigned according to the specificity of the renal disease context, availability of mechanistic validation, and strength of experimental evidence. It does not indicate confirmed clinical efficacy

*ADAM19* a disintegrin and metalloproteinase domain-containing protein 19, *ADSCs* adipose-derived stem cells, *adMSCs* adipose-derived mesenchymal stem cells, *AKI* acute kidney injury, *Akt* protein kinase B, *BMSCs* bone marrow mesenchymal stem cells, *CKD* chronic kidney disease, *EMT* epithelial–mesenchymal transition, *ERK* extracellular signal-regulated kinase, *ET-1* endothelin-1, *EVs* extracellular vesicles, *HDAC1* histone deacetylase 1, *hucMSCs* human umbilical cord mesenchymal stem cells, *hUSCs* human urine-derived stem cells, *IRAK1* interleukin-1 receptor-associated kinase 1, *mTOR* mammalian target of rapamycin, *NF-κB* nuclear factor-κB, *NFAT5* nuclear factor of activated T cells 5, *PFKM* phosphofructokinase, muscle type, *PGAM5* phosphoglycerate mutase family member 5, *RIRI* renal ischemia–reperfusion injury, *RIPK1* receptor-interacting serine/threonine-protein kinase 1, *Sema3A* semaphorin 3A, *STAT1* signal transducer and activator of transcription 1, *STAT5* signal transducer and activator of transcription 5, *TGF-β1* transforming growth factor-β1, *TLR4* Toll-like receptor 4, *TRAF6* tumor necrosis factor receptor-associated factor 6, *VEGFA* vascular endothelial growth factor A, *ZEB2* zinc finger E-box-binding homeobox 2

It is also important to distinguish therapeutic SC-exosomes from urinary or circulating exosomes used for biomarker discovery. Urinary EVs have been proposed as non-invasive sources of renal disease biomarkers, but their clinical translation still requires standardized vesicle isolation, normalization, characterization, and validation in larger disease-specific cohorts [71, 72]. For example, nephrin-positive podocyte-derived urinary EVs and urinary synaptopodin may reflect podocyte injury and glomerular disease activity [73, 74]. In addition, urinary exosomal proteins and RNAs have been investigated in diabetic nephropathy, IgA nephropathy, and renal cancer, including AQP2/AQP5, CCL2 mRNA, urinary exosomal proteins, and exosomal miRNAs [75–78]. These biomarker findings may help clarify disease activity and renal injury patterns, but they should not be regarded as direct evidence for the therapeutic efficacy of SC-exosomes.

**Fig. 2:**
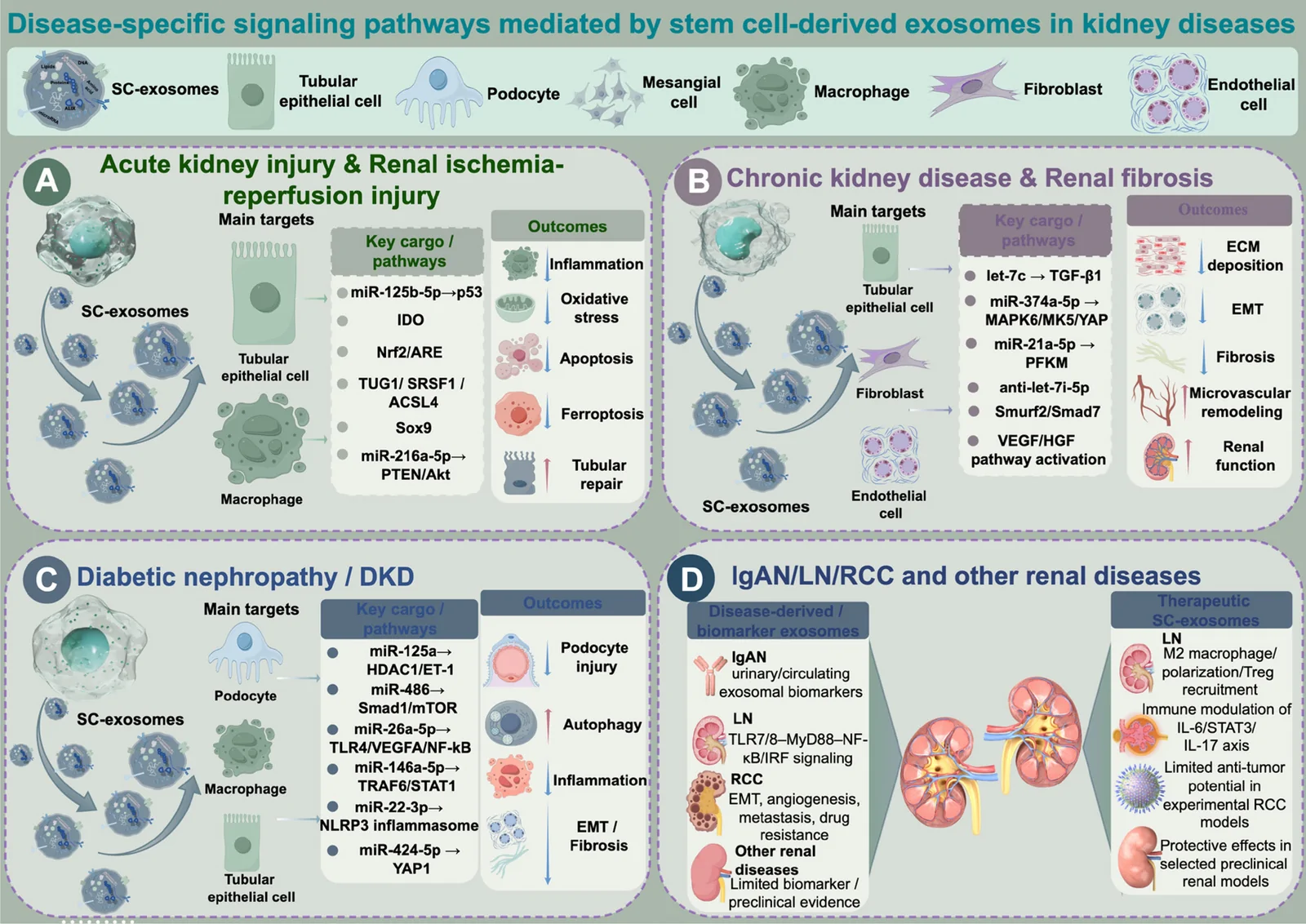
Disease-specific signaling pathways mediated by stem cell-derived exosomes in kidney diseases. The schematic summarizes representative target cells, cargo-related pathways, and renal outcomes associated with SC-exosomes in AKI/RIRI, CKD-associated renal fibrosis, and diabetic nephropathy/DKD. Major mechanisms include anti-inflammatory, anti-apoptotic, anti-oxidative/anti-ferroptotic, and anti-fibrotic effects involving p53, PTEN/Akt, Nrf2/ARE, TGF-β/Smad, NF-κB, NLRP3, and YAP-related signaling. For IgAN, LN, RCC, and other renal diseases, the evidence is more heterogeneous and should be interpreted according to vesicle source and evidence type. Disease-derived or biomarker exosomes should be distinguished from therapeutic SC-exosomes. Current evidence remains predominantly preclinical

The major disease-specific signaling axes through which SC-exosomes regulate renal injury and repair are illustrated in Fig. 2. The schematic highlights the principal target cells, cargo-related pathways, and renal outcomes in AKI/RIRI, CKD-associated renal fibrosis, and DN/DKD, while distinguishing therapeutic SC-exosomes from disease-derived or biomarker exosomes in IgAN, LN, RCC, and other renal diseases.

### SC-exosomes in acute kidney injury (AKI)

During renal hypoxic injury, exosomal miR-374b-5p levels are increased. This miRNA promotes pro-inflammatory M1 macrophage activation by targeting suppressor of cytokine signaling 1 (SOCS1), potentially contributing to kidney disease progression through SOCS1 downregulation [91–93]. In addition, miR-19b-3p and miR-23a, which are present in exosomes released from renal tubular epithelial cells, also contribute to M1 macrophage activation and inflammatory responses. Exosomal miR-19b-3p can further activate M1 macrophages by downregulating SOCS1, triggering NF-κB signaling, and promoting renal tubulointerstitial inflammation [94, 95]. By contrast, hypoxia-pretreated adMSC-exosomes delivered mmu_circ_0001295 and inhibited sepsis-induced kidney injury [69]. MSC-exosomes have also been reported to alleviate AKI by promoting tubular epithelial cell (TEC) regeneration, inhibiting apoptosis, and exerting antioxidant and anti-inflammatory effects. In ischemic AKI, MSC-exosomes may support tubular repair by inhibiting cell-cycle arrest and TEC apoptosis through the miR-125b-5p/p53 signaling pathway [56]. BMSC-exosomes enriched in miR-10a and miR-486 may promote TEC proliferation and reduce AKI-associated renal damage, including blood urea nitrogen (BUN) levels [96]. MSC-exosomes may also exert antioxidant activity. During AKI, they can accumulate in renal tubules and activate the Kelch-like ECH-associated protein 1 (Keap1)/nuclear factor erythroid 2-related factor 2 (Nrf2) signaling pathway, thereby improving TEC mitochondrial function and promoting renal functional recovery [57]. MSC-derived EVs have also been reported to synergize with pulsed focused ultrasound (pFUS) to downregulate heat shock protein 70 (HSP70), NLRP3, IL-1β, and IL-18, thereby ameliorating AKI [58]. In the context of oxidative stress, adMSC-exosomes may upregulate Sox9 expression in tubular epithelium, promote regeneration, attenuate AKI-associated renal dysfunction, and reduce the AKI-to-CKD transition [59]. Urinary or exosome-associated miRNAs, such as miR-494, may serve as candidate diagnostic biomarkers for AKI. However, these biomarker findings should be distinguished from direct therapeutic evidence for SC-exosomes, because the cellular origin, vesicle subtype, and functional relevance of urinary miRNAs may vary across disease contexts. Further validation in standardized clinical cohorts is required before these miRNAs can be applied in routine diagnosis or treatment monitoring. Because renal ischemia–reperfusion injury (RIRI) is one of the most widely studied AKI models in SC-exosome research, RIRI-related evidence is discussed separately in the following section [97, 98].

### SC-exosomes in renal ischemia–reperfusion injury (RIRI)

Renal ischemia–reperfusion injury (RIRI) is a major cause of acute kidney injury (AKI) and is characterized by tubular epithelial cell injury, oxidative stress, mitochondrial dysfunction, inflammatory activation, apoptosis, and, in some settings, ferroptosis [52, 98]. Exosome-mediated intercellular communication has been implicated in both the progression and repair of RIRI. For example, increased exosomal miR-155 may inhibit forkhead box O3a (FoxO3a) and downregulate its downstream apoptosis repressor with caspase recruitment domain (ARC), thereby increasing caspase-1 activation, inflammatory cytokine production, and tubular epithelial cell apoptosis, which may aggravate renal injury [99, 100].

In contrast, stem cell-derived exosomes (SC-exosomes), particularly mesenchymal stem cell-derived exosomes (MSC-exosomes), have shown protective effects in experimental models of RIRI through several complementary mechanisms, including anti-apoptotic, anti-inflammatory, antioxidant, anti-fibrotic, and pro-regenerative effects [101, 102]. First, SC-exosomes may reduce tubular epithelial cell apoptosis and promote tubular repair. MSC-derived EVs can be internalized by injured renal tubular cells and may protect renal function by inhibiting cell death and supporting regenerative responses [103]. Similarly, hucMSC-derived exosomes have been shown to increase miR-21-5p levels in HK-2 cells, thereby suppressing hypoxia/reoxygenation-induced apoptosis and attenuating renal injury in preclinical ischemia–reperfusion/AKI models through the miR-21-5p/TEAD1 axis [104]. hUSC-exosomes enriched in miR-216a-5p have also been reported to suppress phosphatase and tensin homolog (PTEN) and activate the Akt pathway, thereby reducing tubular cell apoptosis and alleviating ischemia–reperfusion-induced renal damage [105].

Second, SC-exosomes may attenuate oxidative stress and mitochondrial injury. For instance, human Wharton’s jelly MSC-derived exosomes (hWJMSC-exosomes) have been shown to activate the Nrf2/antioxidant response element (ARE) pathway, which may enhance antioxidant defense and reduce oxidative injury in RIRI models [60, 106]. Hypoxia-preconditioned adMSC-exosomes may further enhance renal protection by exerting anti-apoptotic, antioxidant, and immunomodulatory effects in injured renal tissue [107, 108]. These findings suggest that the parental cell source and preconditioning status may influence the cargo composition and biological activity of SC-exosomes.

Third, SC-exosomes may modulate ferroptosis and other regulated cell death pathways. hUSC-exosomes enriched in long non-coding RNA taurine-upregulated gene 1 (TUG1) can interact with serine/arginine-rich splicing factor 1 (SRSF1) to regulate acyl-CoA synthetase long-chain family member 4 (ACSL4) mRNA, thereby attenuating ferroptosis in tubular epithelial cells and protecting against ischemia–reperfusion-induced AKI [61]. This mechanism expands the potential role of SC-exosomes beyond conventional anti-inflammatory and anti-apoptotic effects and suggests that regulation of lipid peroxidation and ferroptosis may be an important direction for future studies.

Overall, current evidence indicates that SC-exosomes may alleviate RIRI mainly by reducing tubular apoptosis, suppressing oxidative stress, modulating inflammation, promoting tubular repair, and inhibiting ferroptosis. However, most available data are derived from in vitro studies and animal models, and clinical evidence for SC-exosome-based therapy in RIRI remains lacking. Key translational issues, including optimal exosome source, dose, timing of administration, delivery route, biodistribution, cargo heterogeneity, and long-term safety, require further investigation before SC-exosomes can be considered for clinical application in RIRI [17, 98, 102]. Therefore, SC-exosome-based therapy for RIRI should still be regarded as a promising preclinical strategy rather than an established clinical treatment.

### SC-exosomes in chronic kidney disease (CKD)

Renal tubular atrophy and interstitial fibrosis are common features of chronic kidney disease (CKD) progression. Factors such as transforming growth factor-β1 (TGF-β1) and urinary obstruction can stimulate renal tubular cells to release increased amounts of miR-21-containing exosomes. miR-21 can target the PTEN/Akt signaling pathway and activate fibroblasts, thereby exacerbating renal fibrosis [109]. By contrast, miR-374a-5p carried by MSC-exosomes can inhibit TGF-β1-induced apoptosis of TECs and alleviate renal fibrosis by regulating the MAPK6/MK5/YAP signaling pathway [64]. During hypoxia or ischemia, the level of exosomal miR-150-5p increases, which targets and inhibits *SOCS1* expression, ultimately activating fibroblasts [110]. The sonic hedgehog (Shh) signaling pathway mediates epithelial–mesenchymal transition (EMT) and promotes renal fibrosis. Kidney injury can trigger the release of Shh-containing exosomes from renal tubular epithelial cells, which may further exacerbate fibrosis through a positive feedback mechanism [111]. In addition, exosomal integrins may adhere to inflammatory renal cells and promote inflammatory cell homing through αLβ2 integrin (LFA-1) and α4β1 integrin (VLA-4), thereby contributing to renal inflammation and fibrosis [112]. Intravenous BMSC-exosomes have been shown to reduce renal tubular injury and fibrosis and improve renal function [63, 113]. MSC-exosomes partially retain the anti-inflammatory, antioxidant, anti-apoptotic, anti-fibrotic, and pro-angiogenic effects of MSCs and serve as carriers of bioactive proteins, lipids, nucleic acids, and other molecules, which may attenuate CKD progression in experimental models [14, 62, 63, 114]. Their anti-fibrotic effects are mainly mediated by regulation of fibrosis-related genes. For example, exosomes derived from breast milk mesenchymal stem cells (Br-MSCs) are enriched in anti-fibrotic miR-181, miR-29b, and let-7b, which can attenuate adenine-induced kidney injury and renal fibrosis. These exosomes may also regulate the long non-coding RNA SNHG7 to modulate autophagy in kidney cells, thereby contributing to CKD amelioration in preclinical studies [115]. BMSC-exosomes can attenuate TGF-β1-induced fibrotic responses in renal tubular epithelial cells and reduce renal fibrosis by downregulating fibrotic markers such as α-SMA and collagen I through the Smurf2/Smad7 axis [116, 117]. MSC-derived microvesicles carrying miR-451a have been reported to target the 3′UTR sites of the cell-cycle inhibitors P15INK4b and P19INK4d, thereby relieving cell-cycle arrest, promoting tubular epithelial cell proliferation, and alleviating EMT-associated renal fibrosis in early diabetic kidney injury [118, 119]. Anti-apoptotic effects may also help limit the transition from AKI to CKD. MSC-derived EVs can be enriched with angiogenic factors such as VEGF and HGF, which promote microvessel regeneration, recovery of renal blood flow, and improvement of GFR after transfer to renal tubular cells [68]. MSC-exosomes may also modulate TGF-β-associated responses and promote anti-inflammatory M2-type macrophage polarization, thereby helping to alleviate inflammation and renal fibrosis under certain conditions [120]. hucMSC-exosomes have potential therapeutic relevance in renal failure through modulation of inflammation and oxidative stress [121]. For anti-fibrotic therapy, delivery of anti-let-7i-5p via MSC-exosomes inhibited extracellular matrix (ECM) deposition and EMT, further attenuating renal fibrosis and improving renal function [65].

Although these findings suggest that SC-exosomes may attenuate inflammation, oxidative stress, tubular injury, vascular calcification, and fibrosis in experimental CKD models, their efficacy, optimal dosing strategy, biodistribution, and long-term safety in human CKD remain to be established.

### SC-exosomes in IgA nephropathy (IgAN)

IgA nephropathy (IgAN) is a common glomerular disease characterized by the predominant deposition of immunoglobulin A (IgA) in the mesangial region of the glomerulus, leading to mesangial proliferation, glomerular inflammation, podocyte injury, proteinuria, and progressive renal dysfunction. Exosomes have attracted increasing attention in IgAN because they may participate in intercellular communication within the glomerular microenvironment and may also serve as non-invasive biomarker candidates.

Several urinary or circulating exosomal molecules have been associated with IgAN. For example, elevated levels of miR-144-3p and miR-486-5p have been detected in urinary sediment or EV-associated fractions from patients with IgAN [122]. More recent studies have further identified urinary exosomal miRNA signatures in IgAN, supporting their potential value as non-invasive diagnostic or prognostic biomarkers [123, 124]. In particular, urinary exosomal miR-451a has been reported as a potential non-invasive biomarker for IgAN diagnosis and may reflect tubulointerstitial damage and therapeutic response [125]. However, the functional relevance of miR-144-3p and miR-486-5p in IgAN remains incompletely defined. Current evidence has not fully established whether these miRNAs directly regulate mesangial cell activation, podocyte dysfunction, complement-related injury, or inflammatory signaling in IgAN. Therefore, these miRNAs should be interpreted primarily as disease-associated exosomal miRNAs or candidate biomarkers rather than validated therapeutic cargoes of SC-exosomes.

Other exosomal or immune-related markers have also been investigated in IgAN. B-cell activating factor (BAFF)- and a proliferation-inducing ligand (APRIL)-related signaling is closely associated with B-cell activation and IgAN pathogenesis [126, 127]. In addition, endothelial- or monocyte-derived extracellular vesicles may act on podocytes, promote the secretion of inflammatory mediators, reduce albumin endocytosis, and thereby contribute to proteinuria [128]. Urinary exosomal miRNAs and plasma exosomal interleukin-1 receptor-associated kinase 1 (IRAK1) have also been proposed as potential biomarkers for IgAN diagnosis, disease activity, and therapeutic response [129]. Recent evidence further suggests that B-cell-derived exosomal miR-483-5p may contribute to progressive IgAN by inducing pro-inflammatory changes in renal tubular epithelial cells, providing a mechanistic link between immune dysregulation, exosome-mediated communication, and tubulointerstitial injury [130]. Nevertheless, most of these findings represent biomarker or disease-association evidence and should be distinguished from direct evidence supporting SC-exosome-based therapy.

Compared with the expanding biomarker literature, evidence regarding the therapeutic role of SC-exosomes in IgAN remains limited. One potentially relevant mechanism is the inhibition of inflammatory pathways, particularly the NLRP3 inflammasome, which is involved in renal inflammation and has been implicated in several kidney diseases, including IgAN [54, 131, 132]. SC-exosomes may exert anti-inflammatory and immunomodulatory effects by modulating inflammasome activation, macrophage responses, and glomerular inflammatory signaling. However, IgAN-specific studies directly evaluating SC-exosomes, their cargoes, target cells, and therapeutic efficacy are still insufficient.

Overall, exosomal molecules in IgAN are more strongly supported as emerging biomarker candidates than as established therapeutic targets. Although SC-exosomes may have potential immunomodulatory and anti-inflammatory relevance in IgAN, current evidence remains largely indirect or preclinical. Future studies should clarify the functional roles of IgAN-associated exosomal miRNAs, identify their direct target pathways, distinguish disease-derived exosomes from stem cell-derived exosomes, and validate the therapeutic potential of SC-exosomes in IgAN-specific experimental models and clinical cohorts.

### SC-exosomes in systemic lupus erythematosus and lupus nephritis (SLE/LN)

Systemic lupus erythematosus (SLE) is a systemic autoimmune disease characterized by loss of immune tolerance, production of pathogenic autoantibodies, immune complex formation, and multi-organ inflammation. Renal involvement is one of the most severe manifestations of SLE and may lead to lupus nephritis (LN), which is driven by immune complex deposition, complement activation, inflammatory cell infiltration, podocyte injury, and tubulointerstitial damage [133].

Exosomes may contribute to SLE and LN through the transfer of immunologically active cargoes, including miRNAs, proteins, and nucleic acids. In particular, exosomal miRNAs may interact with Toll-like receptor (TLR)-mediated innate immune signaling. Extracellular miRNAs can be recognized by endosomal TLRs, especially TLR7 and TLR8, thereby activating downstream MyD88-dependent signaling pathways and promoting NF-κB- and interferon regulatory factor-mediated inflammatory responses [134, 135]. This mechanism may enhance type I interferon production, inflammatory cytokine release, dendritic cell activation, and autoreactive B-cell responses, all of which are central to SLE pathogenesis and may contribute to renal inflammation in LN.

In contrast to disease-associated exosomes, SC-exosomes may exert immunomodulatory effects in SLE/LN. BMSC-exosomes have been reported to promote macrophage polarization toward an anti-inflammatory M2 phenotype, reduce inflammatory T-cell infiltration, enhance anti-inflammatory cytokine production, and promote regulatory T cell (Treg) recruitment, thereby attenuating lupus-related renal injury in experimental models [136]. Mechanistically, BMSC-exosome-mediated macrophage reprogramming may enhance efferocytosis, facilitate the clearance of apoptotic debris, and reduce sustained autoantigen exposure, which is particularly relevant to SLE pathogenesis.

Recent evidence further suggests that human umbilical cord mesenchymal stem cell-derived extracellular vesicles (hucMSC-derived EVs) may improve experimental LN by regulating abnormal T- and B-cell responses. In lupus-prone MRL/lpr mice, hucMSC-derived EVs reduced proteinuria, serum creatinine, renal pathological injury, anti-double-stranded DNA immunoglobulin G (anti-dsDNA IgG) levels, and inflammatory cytokine production. These effects were associated with reduced T helper 1 (Th1), T helper 17 (Th17), double-negative T cells, and plasma cells, increased renal Treg cells, and inhibition of the IL-6/signal transducer and activator of transcription 3 (STAT3)/interleukin-17 (IL-17) signaling pathway [137]. These findings indicate that SC-exosomes and related stem cell-derived EVs may modulate both innate and adaptive immune responses in LN.

Nevertheless, current evidence supporting SC-exosome-based therapy for SLE/LN remains largely preclinical [17, 137]. The interaction among exosomes, miRNAs, and TLR signaling is biologically plausible, but the precise exosomal cargoes, recipient immune or renal cells, and disease-specific target pathways require further clarification [134, 135]. Future studies should distinguish pathogenic exosomes derived from immune or renal cells from therapeutic SC-exosomes, identify the specific miRNA-TLR or miRNA-immune-cell axes involved in LN, and validate the safety, dose, delivery route, and long-term immunological effects of SC-exosomes in standardized lupus nephritis models and clinical studies [17].

### SC-exosomes in diabetic nephropathy (DN)

Diabetic nephropathy (DN), also referred to as diabetic kidney disease (DKD), is a major cause of end-stage renal disease. Its progression involves multiple pathological changes, including podocyte injury, mesangial expansion, glomerular basement membrane thickening, tubular epithelial injury, chronic inflammation, epithelial–mesenchymal transition (EMT), and renal fibrosis. During this process, podocytes, mesangial cells, tubular epithelial cells, and infiltrating immune cells can release inflammatory and pro-fibrotic mediators, such as VEGFA, TGF-β, IL-6, TNF-α, and IFN-γ, which further aggravate renal inflammation and functional decline [138, 139].

Several studies have investigated the protective effects of SC-exosomes in DN models. One major mechanism is the suppression of renal inflammation. hucMSC-exosomes enriched in miR-22-3p have been reported to inhibit activation of the NLRP3 inflammasome, thereby reducing podocyte inflammation and renal injury in experimental DN [54]. In addition, miR-146a-5p-modified hucMSCs have been reported to promote M2 macrophage polarization by targeting the TRAF6/STAT1 pathway, leading to reduced inflammatory injury and improved renal function in DN models [53]. Recent evidence also suggests that MSC-exosomes may reduce podocyte inflammation and diabetic kidney injury through modulation of the nucleotide-binding oligomerization domain-containing protein 2 (NOD2) pathway [140].

Podocyte protection is another important aspect of SC-exosome-mediated renal repair in DN. adMSC-exosomal miR-26a-5p has been shown to target TLR4 and suppress VEGFA/NF-κB signaling, thereby reducing podocyte injury and helping preserve glomerular filtration barrier function [106, 141, 142]. Similarly, ADSC-exosomes enriched in miR-486 can inhibit the Smad1/mTOR pathway, promote autophagy flux, reduce podocyte apoptosis, and alleviate renal injury in DN models [80]. These findings suggest that SC-exosomes may act on podocytes through both inflammatory and autophagy-related pathways.

SC-exosomes may also participate in the regulation of EMT and renal fibrosis. hucMSC-exosomes can carry miRNAs, circRNAs, lncRNAs, and proteins that influence the Hedgehog/smoothened (SMO) pathway, thereby reducing tubular EMT and renal fibrosis in DN [66]. MSC-exosomes carrying miR-424-5p have also been reported to target Yes-associated protein 1 (YAP1), suppress DN-induced EMT, and reduce apoptosis in HK-2 cells [67]. In addition, BMSC-exosomes may reduce tubular injury and renal interstitial fibrosis partly through regulation of mTOR-related signaling [65, 141]. These studies indicate that the anti-fibrotic effects of SC-exosomes are likely mediated by multiple pathways rather than a single cargo or target.

It should be noted that not all exosomes in DN are protective. Under diabetic or inflammatory conditions, disease-derived exosomes may contribute to renal injury. For example, tubular epithelial cell-derived exosomes can activate NF-κB signaling in macrophages and promote chronic tubulointerstitial inflammation, suggesting that endogenous renal cell-derived exosomes may act as pathological mediators [23]. In addition, impaired autophagy in adipose tissue may enhance exosome secretion and aggravate insulin resistance and obesity through TLR4/NF-κB signaling, which may indirectly contribute to metabolic stress relevant to DN [143]. These findings highlight the need to distinguish therapeutic SC-exosomes from disease-derived exosomes involved in DN progression.

Exosomal miRNAs have also been explored as candidate biomarkers for DN. Exosomal miR-133b, miR-342, and miR-30a have been identified as potential non-invasive urinary biomarkers for type 2 diabetic nephropathy and are associated with blood pressure, serum creatinine, urinary albumin-to-creatinine ratio, and estimated glomerular filtration rate [144]. Other exosomal miRNAs, including miR-15b-5p, miR-320c, let-7c-5p, miR-21-5p, and miR-30b-5p, have also been proposed for monitoring DN progression and renal injury [145]. However, these findings still require validation in larger and more standardized clinical cohorts.

Taken together, SC-exosomes may influence DN progression by modulating inflammation, macrophage polarization, podocyte injury, autophagy, EMT, and fibrosis. However, most evidence remains based on cell experiments and animal models. Differences in parental cell source, exosomal cargo, isolation methods, dosing strategy, delivery route, biodistribution, and long-term safety remain important barriers to clinical translation [16–18]. Therefore, SC-exosome-based therapy for DN should currently be regarded as a promising experimental strategy rather than an established clinical treatment [17].

### Exosomes and SC-exosomes in renal cancer

Renal cell carcinoma (RCC) is the most common form of kidney cancer and is characterized by a high degree of molecular heterogeneity, metastatic potential, and therapeutic resistance [49, 51]. In RCC, most current evidence on exosomes comes from studies of tumor-derived exosomes or cancer stem cell-derived exosomes rather than stem cell-derived exosomes. Therefore, it is important to distinguish the pathogenic roles of tumor-associated exosomes from the potential therapeutic use of SC-exosomes or engineered EVs [49, 51].

Tumor-derived exosomes can participate in RCC progression by regulating cancer cell proliferation, apoptosis, angiogenesis, immune escape, epithelial–mesenchymal transition (EMT), metastasis, and treatment resistance. For example, miR-9-5p carried by renal cancer cell-derived exosomes can target SOCS4, reduce its inhibitory effect on the JAK/STAT3 pathway, promote cancer cell growth, and inhibit apoptosis [146]. Exosomes may also promote tumor-associated angiogenesis by activating endothelial cells and increasing the release of pro-angiogenic mediators such as VEGF, fibroblast growth factor (FGF), and angiopoietin 1 (ANGPT1) [147, 148]. In addition, exosomal heat shock protein 72 (HSP72) may enhance myeloid-derived suppressor cell (MDSC) activity through STAT3 signaling, thereby contributing to immune suppression within the tumor microenvironment [149]. Cancer stem cell-derived exosomal miR-19b-3p has also been reported to promote metastasis and EMT in clear cell RCC by suppressing PTEN expression [150]. These findings indicate that exosomal miRNAs can function as mediators of tumor progression, although they should be distinguished from therapeutic SC-exosomal cargoes. Exosome-induced EMT may further enhance RCC invasion and metastasis [51, 151, 152]. Recent reviews also support the view that RCC-derived exosomes are closely involved in tumor microenvironment remodeling, immune regulation, metastasis, and drug resistance [51].

Exosomal proteins and miRNAs have also been explored as candidate biomarkers for RCC. Raimondo et al. reported that urinary exosomes from kidney cancer patients showed increased levels of several proteins, including matrix metalloproteinase-9 (MMP9), ceruloplasmin (CP), podocalyxin-like protein (PODXL), carbonic anhydrase IX (CAIX), and dickkopf 4 (DKK4), compared with healthy individuals [77]. Zhang et al. found that serum exosomal miR-210 and miR-1233 were increased in patients with clear cell RCC and decreased after tumor resection, suggesting their potential value for disease monitoring [78]. More recent studies have further emphasized urinary EVs as a non-invasive source of kidney cancer biomarkers, including proteins, miRNAs, mRNAs, and other cargoes [153]. A recent meta-analysis also suggested that exosomal miRNAs may have diagnostic value in RCC, but the included studies were limited in number and heterogeneous in design, indicating that further validation is still needed before clinical application [154].

Compared with tumor-derived exosomes and biomarker studies, direct evidence for SC-exosomes in renal cancer remains limited. One study showed that human liver stem cell-derived EVs inhibited renal cancer stem cell-derived tumor growth in vitro and in vivo, partly through the transfer of anti-tumor miRNAs [155]. This finding suggests that selected stem cell-derived vesicles may have anti-tumor potential in RCC. In addition, engineered extracellular vesicles may serve as delivery platforms for therapeutic molecules, tumor antigens, or immunomodulatory cargoes. However, this possibility should be interpreted cautiously, as exosomes can also participate in immune escape, angiogenesis, EMT, and drug resistance depending on their cellular source and cargo composition [49, 51].

Taken together, exosomes in renal cancer currently have stronger evidence as mediators of tumor biology and candidate liquid-biopsy biomarkers than as established therapeutic agents [49, 51, 153, 154]. SC-exosomes or engineered EVs may provide a future platform for anti-tumor cargo delivery, but their application in RCC requires careful evaluation of cargo specificity, tumor-targeting efficiency, off-target effects, pro-angiogenic or pro-metastatic risks, and long-term safety [51, 155]. At present, SC-exosome- or stem cell-derived EV-based therapy for renal cancer should therefore be regarded as experimental, and further work is needed to separate pathogenic tumor-derived exosomes from therapeutic stem cell-derived or engineered vesicles [51].

### SC-exosomes in other renal diseases

In addition to AKI, CKD, DN, IgAN, lupus nephritis, and renal cancer, exosomes have also been investigated in several other renal conditions, including kidney transplantation, hypertension-associated kidney injury, renal vascular calcification, subtotal nephrectomy, and polycystic kidney disease (PKD). However, the evidence in these settings is generally less mature and should be interpreted according to the source of the exosomes and the type of evidence available. In many cases, the available data involve disease-derived exosomes or urinary EVs as biomarkers rather than direct therapeutic evidence for SC-exosomes [156].

In kidney transplantation, exosomes released by immune cells may carry alloantigens and participate in T-cell activation, which may contribute to acute rejection after transplantation [157]. More recent studies have also discussed EVs as potential non-invasive biomarkers for kidney allograft rejection, because their cargo may reflect immune activation and graft injury [158]. At present, however, their clinical use for rejection monitoring still requires further validation, particularly in larger cohorts and standardized sampling protocols.

In hypertension-associated kidney injury, EVs have been explored both as biomarkers and as potential therapeutic mediators. In hypertension-associated or renovascular kidney injury, MSC-derived EVs have been investigated mainly in preclinical renal artery stenosis models. Adipose tissue-derived MSC-derived EVs improved renal microvascular density and attenuated renal injury in swine with metabolic renovascular disease, whereas donor obesity was later shown to weaken the reparative effect of MSC-derived EVs [68, 159]. More recently, MSC-derived EVs were reported to induce less xenogeneic immune rejection than parental MSCs in a mouse model of renal artery stenosis, supporting further evaluation of their safety in renovascular kidney injury [160]. In experimental renal artery stenosis, adipose-derived MSC exosomes stabilized systolic blood pressure and showed anti-inflammatory effects, suggesting a possible role in vascular and inflammatory modulation [161]. In CKD-associated vascular calcification, BMSC-exosomal miR-381-3p has been reported to target nuclear factor of activated T cells 5 (NFAT5), thereby reducing vascular calcification and apoptosis [88]. These findings are promising, but they remain mainly preclinical and do not yet establish SC-exosomes as a clinical treatment for hypertensive nephropathy.

Exosomes have also been studied in surgical or structural models of kidney injury. In a subtotal nephrectomy model, bone marrow stem cell-derived microvesicles reduced inflammatory cell infiltration and tubular atrophy, suggesting a protective effect against remnant kidney injury and progressive renal damage [162]. This type of evidence supports the renal reparative potential of MSC-derived vesicles, but the optimal cell source, vesicle subtype, dose, and treatment schedule remain unclear.

In PKD, the role of exosomes appears to be more complex. Exosomes produced by cystic renal epithelial cells can promote cyst growth by activating PKD-related signaling pathways, stimulating fibroblasts, and enhancing cytokine production [50]. Urinary exosomal activator of G-protein signaling 3 (AGS3) has also been proposed as a potential biomarker related to PKD pathogenesis [163]. More recent profiling of urinary EV small RNAs in autosomal dominant PKD further supports the potential value of urinary EV cargoes for biomarker discovery and disease monitoring [164]. These findings again highlight the need to distinguish pathogenic disease-derived exosomes from therapeutic SC-exosomes.

Beyond individual diseases, exosomal proteins and RNAs have been investigated as indicators of disease activity, renal injury, and prognosis in several kidney disorders, including AKI, DN, IgAN, lupus nephritis, focal segmental glomerulosclerosis (FSGS), renal fibrosis, hereditary nephropathies, and genitourinary tumors [72, 76]. Recent reviews have similarly emphasized that urinary EVs may provide a non-invasive window into renal injury, while MSC-derived EVs may have immunomodulatory and reparative properties in kidney disease [165]. Nevertheless, most proposed EV biomarkers still require validation in standardized, disease-specific clinical cohorts before routine use.

The therapeutic effects of SC-exosomes may also depend on the parental stem cell source. For example, evidence from DN/DKD models suggests that parental MSC source may influence the cargo composition and dominant biological effects of SC-exosomes. ADSC/adMSC-exosomes and BMSC-exosomes have both shown renoprotective activity in diabetic renal injury models, although direct head-to-head comparisons between these exosome sources remain limited [79, 80, 166]. Differences have also been described between liver-derived MSCs and adMSCs, with stronger immunomodulatory properties reported for liver-derived MSCs and better pro-angiogenic or vascular repair effects reported for adMSCs [167]. These observations suggest that parental cell selection may influence the cargo composition and biological activity of exosomes. For renal diseases, however, source selection should be guided by disease-specific mechanisms, target cells, safety considerations, and reproducible functional assays rather than by a general assumption that one stem cell source is superior to another.

These findings suggest that evidence in less-studied renal diseases is still heterogeneous, with some studies supporting biomarker discovery and others indicating possible reparative effects of SC-exosomes in preclinical models. In kidney transplantation and PKD, current studies mainly support pathogenic or biomarker-related roles of disease-derived and urinary EVs, whereas direct therapeutic evidence for SC-exosomes remains limited. In hypertension-associated kidney injury, renal vascular calcification, and subtotal nephrectomy models, MSC-derived EVs have shown reparative or anti-inflammatory effects in preclinical settings, but their clinical relevance remains uncertain. Future studies should separate disease-derived exosomes from therapeutic SC-exosomes, define the relevant cargoes and target pathways, and validate source-specific effects in appropriate renal disease models before clinical translation is considered.

**Fig. 3:**
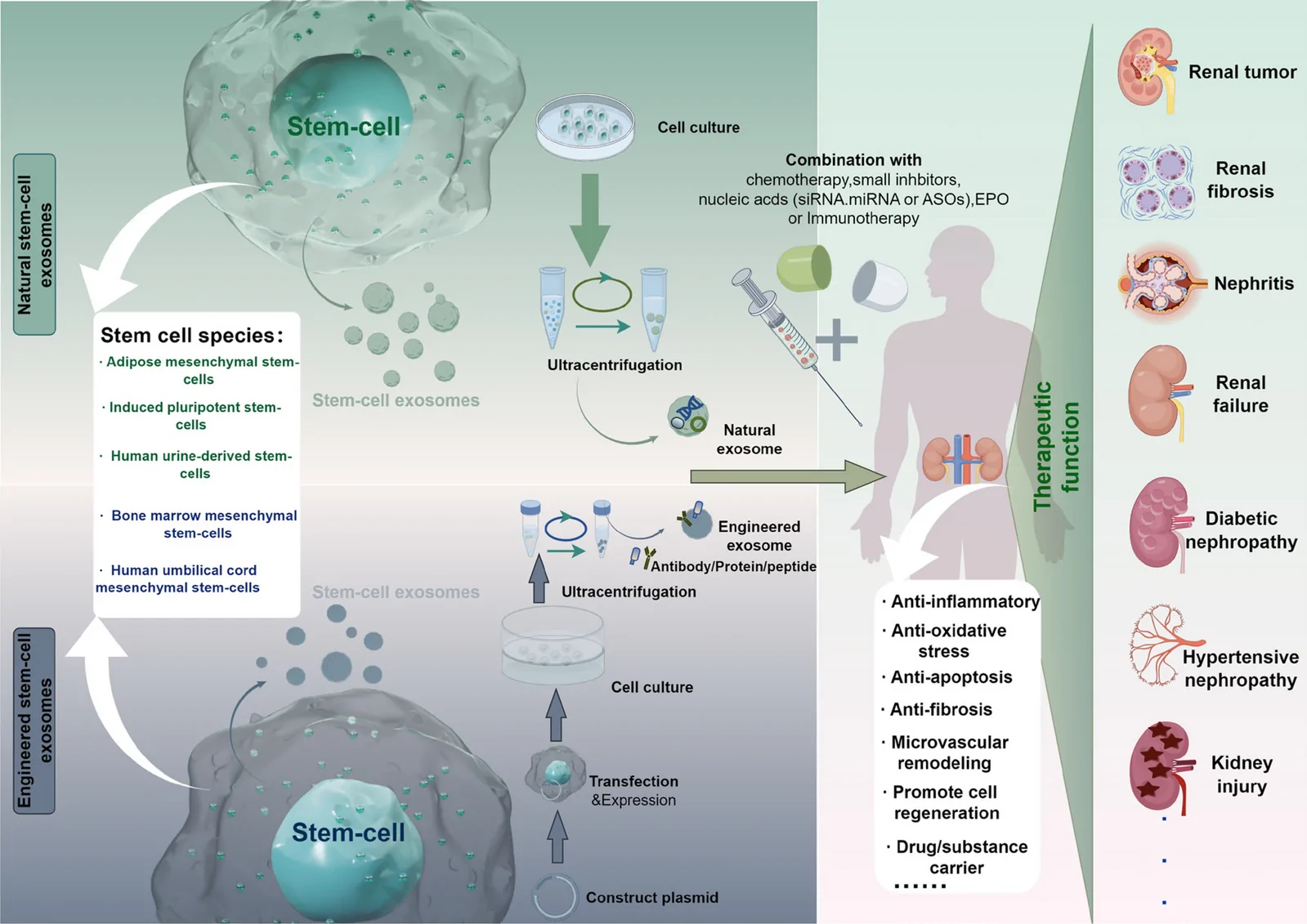
Natural stem cell-derived exosomes versus engineered stem cell-derived exosomes and their therapeutic role in kidney disease. Stem cell sources for exosomes include adipose mesenchymal stem cells, human urine-derived stem cells, bone marrow mesenchymal stem cells, human umbilical cord mesenchymal stem cells, induced pluripotent stem cells, and other stem cell types. Natural stem cell-derived exosomes can be extracted after cell culture and ultracentrifugation; engineered stem cell-derived exosomes can be artificially constructed after plasmid construction, transfection, cell culture, ultracentrifugation, and drug/antibody loading. They have been investigated in preclinical models of several renal diseases (e.g., renal tumors, renal fibrosis, nephritis, renal failure, diabetic nephropathy) through anti-inflammatory, anti-oxidative stress, anti-apoptotic, anti-fibrotic, microvascular remodeling, and cellular regeneration effects. In addition, SC-exosomes may be combined with chemotherapy, erythropoietin, inhibitory molecules, nucleic acid-based strategies, or immunotherapy in selected experimental settings

Overall, SC-exosomes may play an important role in intercellular communication among glomerular endothelial cells, tubular epithelial cells, and podocytes during the progression of renal diseases, and these interactions may contribute to attenuating podocyte injury and tubular damage [54, 56, 80]. SC-exosomes have also shown therapeutic potential in various renal disease models through anti-inflammatory, antioxidant, anti-apoptotic, anti-fibrotic, and microvascular remodeling mechanisms, and they may serve as carriers for therapeutic molecules or drugs (Fig. 3). To provide a clearer cross-disease synthesis, we summarized the major exosome sources, representative cargoes or markers, mechanisms, evidence types, and key translational limitations across renal diseases in Table 3. This summary distinguishes disease-derived exosomes, urinary or circulating exosomes used as biomarkers, and therapeutic SC-exosomes where appropriate, because these vesicle populations may have different biological roles and translational implications.

**Table 3:** Summary of exosome/EV sources, representative cargoes, mechanisms, evidence types, and translational limitations across renal diseases

Disease context	Exosome/EV source	Representative cargoes or markers	Main mechanisms or biological roles	Evidence type	Key limitations and translational perspectives	Representative references
Acute kidney injury (AKI)	Renal tubular epithelial cell-derived exosomes, hypoxia-pretreated adMSC-exosomes, MSC-exosomes, MSC-derived EVs, and miRNA-engineered MSC-derived EVs	miR-374b-5p, miR-19b-3p, miR-23a, mmu_circ_0001295, miR-125b-5p, miR-10a, miR-486, HSP70-related cargoes, and Sox9-related signals	Disease-derived tubular exosomes may promote M1 macrophage activation and inflammatory injury, whereas SC-exosomes may reduce tubular epithelial apoptosis, suppress inflammation, improve mitochondrial function, inhibit NLRP3-related injury, and promote tubular repair and regeneration	Mainly cell experiments and animal models	Pathogenic disease-derived exosomes and therapeutic SC-exosomes should be clearly distinguished; clinical evidence remains limited; model heterogeneity, dose, timing, delivery route, and biodistribution require further clarification	[56–59, 69, 91–97]
Renal ischemia–reperfusion injury (RIRI)	BMSC-exosomes, hucMSC-exosomes, adMSC-exosomes, hUSC-exosomes, hWJMSC-exosomes, and MSC-derived EVs	miR-155, miR-21-5p, miR-216a-5p, lncRNA TUG1, miR-19b, and Nrf2/ARE-related antioxidant cargoes	Disease-associated exosomal miR-155 may contribute to apoptotic and inflammatory injury, whereas therapeutic SC-exosomes may reduce tubular apoptosis, suppress oxidative stress and inflammation, activate PTEN/Akt and Nrf2/ARE-related pathways, promote tubular repair, and attenuate ferroptosis through the TUG1/SRSF1/ACSL4 axis	Mainly in vitro studies and animal RIRI models	Clinical evidence is lacking; optimal exosome source, administration window, dose, delivery route, biodistribution, and long-term safety remain unresolved	[60, 61, 98, 99, 101–103, 105, 108, 168]
Chronic kidney disease and renal fibrosis	Renal tubular epithelial cell-derived exosomes, MSC-exosomes, BMSC-exosomes, breast milk MSC-derived exosomes, MSC-derived EVs or microvesicles, macrophage-derived microvesicles, and engineered MSC-exosomes	let-7c, miR-21/miR-21a-5p, miR-150-5p, miR-374a-5p, miR-451a, miR-335-5p, anti-let-7i-5p, TGF-β1-containing cargoes, CCL2 mRNA, and fibrosis-associated proteins	SC-exosomes may attenuate renal fibrosis by regulating TGF-β/Smad-related signaling, MAPK6/MK5/YAP, PFKM-mediated glycolysis, ADAM19-related signaling, autophagy, inflammation, EMT, and extracellular matrix deposition; disease-derived tubular exosomes may also activate fibroblasts and promote inflammation or fibrosis	Mainly mechanistic cell studies and animal fibrosis models	Fibrosis models differ substantially; anti-fibrotic efficacy, disease specificity, durability of response, and standardized potency assays remain to be validated	[62–65, 90, 109–112, 114–116, 118, 156, 169–173]
Diabetic nephropathy / diabetic kidney disease	hucMSC-exosomes, adMSC-exosomes, hADSC-exosomes, hUSC-exosomes, BMSC-exosomes, MSC-exosomes, and urinary exosomes	miR-125a, miR-486, miR-26a-5p, miR-215-5p, miR-16-5p, miR-146a-5p, miR-22-3p, miR-424-5p, AQP2, AQP5, urinary miR-133b, miR-342, miR-30a, and other exosomal miRNAs	SC-exosomes may suppress NLRP3- and NOD2-related inflammation, promote M2 macrophage polarization through TRAF6/STAT1, protect podocytes through HDAC1/ET-1, TLR4/NF-κB, Smad1/mTOR, and Nrf2/Keap1-related pathways, regulate autophagy, inhibit EMT, and reduce renal fibrosis; urinary exosomal proteins and miRNAs may serve as candidate biomarkers	Mainly in vitro and animal DN/DKD models; limited clinical-sample biomarker studies	Therapeutic SC-exosomes should be distinguished from disease-derived or urinary biomarker exosomes; clinical validation of both biomarkers and therapeutic efficacy remains insufficient	[53, 54, 66, 67, 75, 79–83, 85, 86, 138–141, 143–145, 174]
IgA nephropathy (IgAN)	Urinary exosomes, circulating exosomes, EV-associated fractions, monocyte- and endothelial-derived microparticles, B-cell-derived exosomes, and limited indirect evidence for SC-exosomes	miR-144-3p, miR-486-5p, urinary exosomal miRNA signatures, miR-451a, miR-483-5p, CCL2 mRNA, IRAK1, and NLRP3-related inflammatory markers	Exosomal cargoes may reflect glomerular inflammation, podocyte injury, tubulointerstitial damage, disease activity, treatment response, and progressive kidney function loss; SC-exosomes may have indirect anti-inflammatory relevance, but IgAN-specific therapeutic evidence remains limited	Mainly clinical-sample biomarker studies and disease-association studies; limited indirect evidence for SC-exosome therapy	Functional roles of IgAN-associated exosomal miRNAs remain incompletely defined; direct SC-exosome studies, target-cell validation, and disease-specific therapeutic models are still needed	[54, 76, 122–125, 128–132]
Lupus nephritis (LN)/ systemic lupus erythematosus	Disease-associated immune exosomes, BMSC-exosomes, and hucMSC-derived EVs	Exosomal miRNAs, TLR-related nucleic acid cargoes, immune-regulatory cargoes	Disease-associated exosomal nucleic acids may activate TLR7/TLR8-MyD88-NF-κB/IRF-related innate immune signaling; SC-exosomes may promote M2 macrophage polarization, enhance efferocytosis, increase Treg recruitment, and regulate Th1/Th17/Treg imbalance, B-cell responses, and IL-6/STAT3/IL-17 signaling	Mechanistic immune studies and animal models; limited clinical translation	Pathogenic immune-cell-derived exosomes and therapeutic SC-exosomes should be separated; specific miRNA-TLR axes, recipient immune or renal cells, dose, route, and long-term immunological safety require further study	[133–137]
CKD-associated vascular calcification	BMSC-exosomes	miR-381-3p, NFAT5-related pathway	BMSC-exosomal miR-381-3p may alleviate vascular calcification and apoptosis by targeting NFAT5-related signaling	Preclinical mechanistic evidence	Clinical relevance to CKD-associated vascular calcification requires further validation in disease-specific models and patient cohorts	[88]
Renal cell carcinoma (RCC)	Tumor-derived exosomes, cancer stem cell-derived exosomes, urinary or serum exosomes, human liver stem cell-derived EVs, and limited engineered EV evidence	miR-9-5p, miR-19b-3p, miR-210, miR-1233, MMP9, CP, PODXL, CAIX, DKK4, HSP72, and tumor-associated EV cargoes	Tumor-derived exosomes may promote proliferation, angiogenesis, immune escape, EMT, metastasis, and drug resistance; exosomal proteins and miRNAs may serve as candidate liquid-biopsy biomarkers; selected stem cell-derived or engineered EVs may have experimental anti-tumor potential	Tumor biology studies, biomarker studies, and limited preclinical evidence for stem cell-derived or engineered EVs	Tumor-derived exosomes are often pathogenic; therapeutic SC-exosome or engineered EV use in RCC remains experimental and requires careful assessment of cargo specificity, tumor targeting, off-target effects, pro-angiogenic or pro-metastatic risk, and long-term safety	[51, 71, 77, 78, 146, 150, 152–155, 173, 175, 176]
Kidney transplantation and allograft rejection	Immune cell-derived exosomes, urinary EVs, circulating EVs, and donor- or recipient-cell-derived vesicles	Alloantigens, immune-related proteins and RNAs, graft injury-associated EV cargoes	Exosomes may mediate alloantigen transfer and T-cell allorecognition; urinary or circulating EV cargoes may reflect graft injury, immune activation, and allograft rejection	Mechanistic immune studies and clinical biomarker studies	Clinical use for rejection monitoring requires standardized sampling, EV isolation and characterization, multicenter validation, and clearer interpretation of graft-specific EV cargo changes	[157, 158]
Hypertension-associated or renovascular kidney injury	MSC-derived EVs, adMSC-exosomes, BMSC-exosomes	Microvascular repair-related cargoes, immunomodulatory cargoes, angiogenesis-related cargoes	MSC-derived EVs may improve renal microvascular density, attenuate inflammatory injury, support vascular repair, and reduce renal damage in metabolic or chronic renovascular disease models; adipose-derived MSC exosomes/EVs may stabilize systolic blood pressure and modulate renal inflammation in experimental renal artery stenosis	Mainly preclinical animal studies, including large-animal models	Effects may depend on disease stage, metabolic status, obesity, parental cell source, and immune response to administered cells or EVs	[68, 132, 160, 161, 177]
Subtotal nephrectomy / remnant kidney injury	Bone marrow stem cell-derived microvesicles	Reparative and anti-inflammatory vesicle cargoes	May reduce inflammatory cell infiltration and tubular atrophy, suggesting a protective role against progressive remnant kidney injury	Preclinical remnant kidney model	Optimal vesicle subtype, source, dose, timing, and long-term efficacy remain uncertain	[162]
Polycystic kidney disease (PKD)	Cystic renal epithelial cell-derived exosomes, urinary exosomes, urinary EVs	AGS3, urinary EV small RNAs, disease-associated proteins and RNAs	Disease-derived exosomes may promote cyst growth, fibroblast activation, and cytokine production; urinary EV cargoes may provide biomarker information for disease monitoring and pathophysiological studies	Disease-derived EV studies and clinical-sample biomarker studies	Pathogenic disease-derived EVs should be separated from therapeutic SC-exosomes; evidence for SC-exosome therapy in PKD remains insufficient	[50, 163, 164]
Cross-disease biomarker and translational applications	Urinary EVs, circulating EVs, disease-derived exosomes, MSC-derived EVs, and SC-exosomes	Exosomal proteins, miRNAs, mRNAs, small RNAs, and other regulatory cargoes	EV cargoes may reflect renal injury, inflammation, fibrosis, disease activity, or prognosis; MSC-derived EVs may show immunomodulatory, reparative, or anti-fibrotic effects across different renal disease models	Biomarker studies, disease-association studies, and preclinical MSC-EV studies	Most proposed biomarkers require large standardized cohorts; therapeutic applications require consistent isolation, characterization, potency assays, cargo validation, and disease-specific functional testing	[18, 19, 71, 72, 89, 145, 165, 173, 178]
Engineered and production-optimized SC-exosomes	Engineered MSC-exosomes, surface-modified EVs, drug-loaded EVs, immortalized MSC-derived exosomes, and culture-optimized MSC-derived EVs	Anti-let-7i-5p, therapeutic miRNAs or antagomirs, targeting peptides, LAMP-2B fusion constructs, drug-loaded cargoes, optimized protein cargoes	Engineering strategies may improve cargo loading, tissue targeting or kidney-relevant anti-fibrotic delivery, tissue repair, and scalable exosome production; immortalized or culture-optimized MSC systems may help reduce donor variability and improve production consistency	Preclinical engineering studies and translational methodological studies	Targeting specificity, off-target biodistribution, manufacturing consistency, genetic stability, potency, storage, regulatory standards, and long-term safety require validation before clinical translation	[14, 65, 179–190]

This table summarizes representative evidence rather than providing an exhaustive list of all studies. Disease-derived exosomes or EVs, urinary or circulating vesicles used as biomarkers, and therapeutic SC-exosomes or related stem cell-derived EVs are listed separately where relevant, because these vesicle populations may differ in cellular origin, cargo composition, biological function, and translational implications

*ACSL4* acyl-CoA synthetase long-chain family member 4, *ADSCs* adipose-derived stem cells, *adMSCs* adipose-derived mesenchymal stem cells, *adMSC-exosomes* adipose-derived mesenchymal stem cell-derived exosomes, *AGS3* activator of G-protein signaling 3, *AKI* acute kidney injury, *ANGPT1* angiopoietin 1, *AQP2* aquaporin 2, *AQP5* aquaporin 5, *ARE* antioxidant response element, *BMSCs* bone marrow mesenchymal stem cells, *BMSC-exosomes* bone marrow mesenchymal stem cell-derived exosomes, *CAIX* carbonic anhydrase IX, *CCL2* C–C motif chemokine ligand 2, *CKD* chronic kidney disease, *CP* ceruloplasmin, *DKK4* dickkopf 4, *DN* diabetic nephropathy, *DKD* diabetic kidney disease, *ECM* extracellular matrix, *EMT* epithelial–mesenchymal transition, *EVs* extracellular vesicles, *FGF* fibroblast growth factor, *HGF* hepatocyte growth factor, *HSP70* heat shock protein 70, *HSP72* heat shock protein 72, *hucMSCs* human umbilical cord mesenchymal stem cells, *hucMSC-exosomes* human umbilical cord mesenchymal stem cell-derived exosomes, *hucMSC-derived EVs* human umbilical cord mesenchymal stem cell-derived extracellular vesicles, *hUSCs* human urine-derived stem cells, *hUSC-exosomes* human urine-derived stem cell-derived exosomes, *hWJMSCs* human Wharton’s jelly mesenchymal stem cells, *hWJMSC-exosomes* human Wharton’s jelly mesenchymal stem cell-derived exosomes, *IgAN* IgA nephropathy, *IRAK1* interleukin-1 receptor-associated kinase 1, *IRF* interferon regulatory factor, *JAK* Janus kinase, *LAMP-2B* lysosome-associated membrane glycoprotein 2B, *LN* lupus nephritis, *lncRNA* long non-coding RNA, *MDSC* myeloid-derived suppressor cell, *miRNAs* microRNAs, *MMP9* matrix metalloproteinase-9, *mRNAs* messenger RNAs, *MSCs* mesenchymal stem cells, *MSC-exosomes* mesenchymal stem cell-derived exosomes, *MSC-derived EVs* mesenchymal stem cell-derived extracellular vesicles, *MyD88* myeloid differentiation primary response 88, *NFAT5* nuclear factor of activated T cells 5, *NF-κB* nuclear factor-κB, *NLRP3* NOD-like receptor family pyrin domain containing 3, *NOD2* nucleotide-binding oligomerization domain-containing protein 2, *Nrf2* nuclear factor erythroid 2-related factor 2, *PFKM* phosphofructokinase, muscle type, *PKD* polycystic kidney disease, *PODXL* podocalyxin-like protein, *PTEN* phosphatase and tensin homolog, *RCC* renal cell carcinoma, *RIRI* renal ischemia–reperfusion injury, *SC-exosomes* stem cell-derived exosomes, *SMO* smoothened, *Sox9* SRY-box transcription factor 9, *SRSF1* serine/arginine-rich splicing factor 1, *STAT1* signal transducer and activator of transcription 1, *STAT3* signal transducer and activator of transcription 3, *TECs* tubular epithelial cells, *TGF-β1* transforming growth factor-β1, *Th1* T helper 1, *Th17* T helper 17, *TLR* Toll-like receptor; *Treg* regulatory T cell, *TUG1* taurine-upregulated gene 1, *VEGF* vascular endothelial growth factor, *VEGFA* vascular endothelial growth factor A, *YAP* Yes-associated protein

## Preclinical evidence, clinical translation, and engineering strategies

SC-exosomes have been investigated in kidney diseases mainly through preclinical cell-based and animal studies. These vesicles can transfer proteins, mRNAs, miRNAs, and other regulatory molecules to recipient cells and may influence inflammation, apoptosis, oxidative stress, fibrosis, and tissue repair. Compared with direct stem cell transplantation, SC-exosomes are being investigated as a cell-free approach, but their therapeutic value in renal diseases remains largely experimental and requires further validation [17, 165].

### Preclinical evidence from cell and animal models

Most evidence supporting SC-exosomes in renal diseases comes from in vitro studies and animal models. In RIRI models, MSC-exosomes have been reported to reduce renal inflammation, promote tubular epithelial cell repair, and limit fibrotic responses [17]. For example, indoleamine 2,3-dioxygenase (IDO) carried by MSC-exosomes accelerated renal recovery in mice with ischemia–reperfusion injury, reduced M1 macrophage infiltration, attenuated renal inflammation, and promoted M2 macrophage polarization [55].

Other experimental studies have suggested that breast milk MSC-exosomes may reduce serum uric acid, creatinine, and urea levels in adenine-induced nephropathy by regulating the miR-34a/SNHG7/AMPK/ULK1-Akt/mTOR axis and improving antioxidant responses [115]. MSC-exosomes may also attenuate renal fibrosis through autophagy- and fibrotic signaling-related pathways, while adipose-derived stem cell exosomes have been reported to reduce oxidative stress through activation of the Keap1/Nrf2 pathway in diabetic nephropathy [115, 174]. In addition, several miRNAs, including miR-29b, miR-181, and let-7b, have been implicated in SC-exosome-mediated renal protection [115]. These findings indicate that SC-exosomes often act through multiple cargoes and pathways rather than through a single mechanism.

Induced pluripotent stem cell-derived exosomes have also been studied in experimental kidney injury. Intravenous administration of iPSC-exosomes improved renal function and reduced renal fibrosis in mouse models, suggesting that non-MSC stem cell sources may also have renal reparative potential [191–193]. Nevertheless, the effects of SC-exosomes vary according to the parental cell type, injury model, route of administration, and disease stage. Some exosomal miRNAs may also exert pathogenic effects in renal disease; for example, exosomal miR-150-5p has been reported to promote renal fibroblast activation and fibrosis [110]. Therefore, disease-derived exosomes and therapeutic SC-exosomes should be interpreted separately.

Combination strategies have also been explored in preclinical settings. Microvesicles derived from erythropoietin (EPO)-treated MSCs showed stronger renoprotective and anti-fibrotic effects than microvesicles derived from untreated MSCs in experimental CKD models [194]. Such findings suggest that SC-exosomes may be combined with preconditioning or adjunctive therapeutic strategies, but these strategies remain at an early experimental stage.

### Clinical translation and biomarker-related evidence

Clinical translation of SC-exosomes in kidney disease remains limited. Preclinical studies suggest that hucMSC-exosomes may attenuate renal fibrosis by delivering casein kinase 1δ (CK1δ) and β-transducin repeat-containing protein (β-TrCP) to promote YAP ubiquitination and degradation, supporting their potential relevance as a cell-free therapeutic strategy for CKD-associated renal fibrosis [191]. In addition, preliminary clinical evidence involving hucMSC-derived EVs has suggested potential safety and renal functional improvement in patients with stage 3–4 CKD, although these findings require cautious interpretation and further validation [195]. At present, most support for SC-exosome therapy still comes from animal models, and well-designed clinical trials with standardized dose, route, follow-up duration, and safety evaluation are needed.

Exosomes have also been studied as biomarkers in renal diseases. Urinary exosomal proteins may reflect changes in renal tissue more closely than total urinary proteins in some settings and have been proposed as non-invasive biomarkers [196]. In diabetic nephropathy, urinary exosomal AQP5 and AQP2 were increased in a small clinical cohort and may have potential value for monitoring disease progression [75]. In IgA nephropathy, elevated urinary exosomal CCL2 mRNA has been associated with active renal injury and deterioration of renal function [76]. These findings support the biomarker potential of urinary exosomes, but they should not be regarded as evidence for therapeutic efficacy of SC-exosomes.

Thus, clinical translation currently involves two related but distinct directions: the use of urinary or circulating exosomes as diagnostic or prognostic biomarkers, and the development of SC-exosomes as therapeutic agents. Both directions require larger clinical cohorts, standardized exosome isolation and characterization, and reproducible normalization strategies [18, 165].

### Engineering strategies and drug delivery approaches

Because of their lipid bilayer structure, biocompatibility, and ability to carry biological cargoes, exosomes have been explored as natural drug delivery vehicles [31]. In cancer research, exosomes engineered to carry glycosylphosphatidylinositol (GPI)-anchored interleukin-12 (IL-12) enhanced antitumor effects, illustrating the broader feasibility of exosome-based cargo delivery [197]. In renal disease models, MSC-exosomes can also be engineered or modified to deliver functional molecules involved in tissue repair, inflammation control, and fibrosis regulation [14, 190]. For example, MSC-exosomes loaded with anti-let-7i-5p oligonucleotides reduced renal fibrosis and improved renal function in a UUO model [65].

Several cargoes and pathways have been explored in this context. MSC-exosomes may deliver or regulate molecules such as Oct-4, SP1, MFG-E8, Sox9, SIRT1, mTOR-related signals, and YAP-associated pathways, thereby affecting renal fibrosis, inflammation, oxidative stress, apoptosis, and tubular repair [118]. In addition, exosome loading can be achieved through direct loading, parental cell-mediated loading, or membrane encapsulation [180, 181]. Other loading methods include incubation, transfection, sonication, electroporation, in situ synthesis, and ligand-receptor-based approaches [179, 182, 183].

Targeted engineering may further improve the delivery efficiency of exosomes. For example, Jin et al. transfected MSCs with anti-let-7i-5p and isolated exosomes for targeted delivery of anti-let-7i-5p oligonucleotides, which improved renal function and reduced renal fibrosis in mice [65]. Surface modification is another strategy to enhance tissue targeting. Although exosomes derived from commonly used producer cell lines, such as human embryonic kidney 293 (HEK293) cells, may lack natural renal homing capacity, surface engineering may help improve targeting specificity [184, 198]. LAMP-2B is commonly used as an exosomal membrane scaffold to display targeting peptides. For example, E7-Exo was generated by fusing the MSC-binding peptide E7 with LAMP-2B, thereby improving exosome targeting ability [185]. Recent reviews of engineered EVs in CKD also emphasize the importance of cargo loading, surface modification, and kidney-targeted delivery for future therapeutic development [190].

Another strategy is to improve the scalability and consistency of exosome production. Primary MSCs have a finite proliferative lifespan and may undergo senescence during prolonged expansion, which can affect the regenerative properties of MSCs and their derived EVs [199, 200]. In addition, donor-related heterogeneity, including donor age, health status, tissue source, and culture conditions, can influence MSC-derived EVs yield, cargo composition, and functional potency [201, 202]. Immortalized MSCs have therefore been explored as a more stable source for exosome production. Exosomes derived from conditionally immortalized MSCs may help overcome some limitations of primary MSCs, while maintaining exosome production and functional activity [186, 187]. However, immortalized cell sources require careful evaluation of genetic stability, safety, and regulatory acceptability before clinical use.

### Challenges for clinical translation

Several unresolved issues continue to limit the clinical translation of SC-exosomes in renal diseases. First, exosome heterogeneity remains a major barrier to reproducible therapeutic development. The yield, cargo composition, and biological activity of SC-exosomes may vary according to parental cell source, donor characteristics, tissue origin, culture conditions, passage number, preconditioning strategy, isolation method, and storage conditions [201]. Such variability makes it difficult to compare results across studies and to define consistent quality attributes. Therefore, future studies should provide a clearer description of the parental cell source, vesicle subtype, isolation procedure, characterization method, and functional potency assays. The Minimal Information for Studies of Extracellular Vesicles 2023 (MISEV2023) recommendations also emphasize the importance of standardized reporting of EV source, separation strategy, characterization, functional analysis, and experimental controls [18].

Large-scale production and quality control are also important obstacles. Addressing large-scale production, purification of exosomes, batch-to-batch variability, and identification of functional exosome subpopulations is crucial for achieving the clinical translation of exosome-based therapies [17, 203]. Recent advances in three-dimensional culture systems, bioreactors, cell preconditioning, genetic modification, and EV engineering may improve EV yield or cargo loading, but these approaches may also alter vesicle composition and biological function [204]. Thus, scalable manufacturing should be accompanied by reproducible release criteria, validated potency assays, sterility testing, storage stability assessment, and long-term quality control.

Delivery efficiency is another unresolved issue. SC-exosomes can be administered through several routes, including intravenous, intraperitoneal, intranasal, and local administration [179]. However, systemic administration does not necessarily ensure efficient kidney-specific delivery. Off-target biodistribution, rapid clearance, uptake by the mononuclear phagocyte system, and limited retention in injured renal compartments may reduce therapeutic efficacy. Engineering strategies, including surface modification, ligand-mediated targeting, and cargo loading, may improve delivery efficiency and tissue specificity, but they also introduce additional concerns related to vesicle stability, immunogenicity, manufacturing reproducibility, and regulatory evaluation [190].

Dose optimization and treatment timing require further clarification. The therapeutic effects of SC-exosomes may depend on disease stage, severity of renal injury, route of administration, dosing frequency, and the interval between injury and treatment. One study suggested that the effective duration of action of stem cell-derived exosomes may be within 48 h [178], indicating that administration timing may influence therapeutic outcome. However, optimal dose, dosing frequency, treatment window, and repeated-administration strategies have not been standardized across renal disease models. Future studies should therefore include dose–response analyses, biodistribution studies, and longer follow-up periods.

Safety concerns also require careful assessment. Although SC-exosomes are generally considered less tumorigenic than transplanted stem cells, their biological effects are highly context-dependent. SC-exosomes may exert reparative, anti-inflammatory, or anti-fibrotic effects in some models, whereas disease-derived exosomes may promote inflammation, fibrosis, cyst progression, or tumor progression depending on their cellular origin and cargo composition [165]. Therefore, future studies should define the parental cell source, vesicle subtype, cargo composition, target cells, and disease stage more precisely [18]. Immune responses, off-target accumulation, unwanted pro-fibrotic or pro-angiogenic effects, repeated-administration risks, and long-term toxicity should be systematically evaluated before clinical translation.

Taken together, SC-exosomes represent a promising but still developing strategy for renal disease research. Current evidence supports their potential roles in preclinical models, biomarker discovery, and engineered delivery systems. Their nanoscale structure, biological cargo, and relatively low immunogenicity make them attractive candidates for cell-free therapy and targeted delivery. In particular, engineered exosomes may improve cargo loading and tissue targeting, while immortalized MSC-derived exosomes may help address some limitations related to donor variability, cell senescence, and production consistency. However, these approaches remain at an early stage. Before SC-exosomes can be translated into routine clinical use, further studies are needed to establish standardized manufacturing, validated potency assays, optimized dosing and delivery strategies, long-term safety data, and well-designed clinical trials.

## Discussion

As a cell-free therapeutic strategy under active investigation, SC-exosomes have attracted increasing attention in renal disease research. They may retain part of the regenerative and reparative properties of parental stem cells while partly avoiding some cell-associated risks of direct stem cell transplantation, such as poor cell survival, limited engraftment, immunogenicity, and tumorigenic risk. As nanoscale delivery vehicles, exosomes may also deliver therapeutic molecules to injured renal tissue and support the development of more targeted therapeutic approaches. Engineered exosomes may further improve the translational utility of natural exosomes because of their relatively low toxicity, high stability, prolonged in vivo circulation, modifiable surface properties, and capacity for targeted intracellular cargo delivery [189]. Nevertheless, most evidence supporting the therapeutic application of SC-exosomes in renal diseases remains preclinical, and their clinical efficacy and long-term safety require further validation.

In the pathophysiology of kidney disease, exosomes are involved in intercellular communication, cargo transport to specific target cells, and regulation of biological functions in recipient cells. In experimental renal disease models, SC-exosomes have been reported to promote cell proliferation and angiogenesis, inhibit inflammation and fibrosis, and reduce apoptosis and epithelial–mesenchymal transition (EMT). Although the use of exosomes as biomarkers and therapeutic tools is still at an early stage, their potential value in the diagnosis, disease monitoring, and therapeutic development of renal diseases warrants further investigation.

However, the clinical application of SC-exosomes still faces several challenges. At present, standardized criteria for exosome purification, quantification, characterization, and quality control remain insufficient, which limits their broader clinical application. Despite progress in preclinical studies, human studies remain limited, and further clinical trials are needed to confirm the safety and efficacy of exosome-based therapies. Exosome heterogeneity, including differences in size, markers, cargo composition, and biological function, is another key factor hindering clinical translation. This heterogeneity requires further investigation of specific exosomal molecules and functional subpopulations to better clarify how exosomes regulate disease-related cellular processes and to improve the targeting of exosome-based therapies.

Given these challenges, future research should focus on standardized SC-exosome production, scalable purification techniques, and improved therapeutic safety and product quality. Three-dimensional culture systems have been shown to increase exosome yield and enhance the functional properties of MSC-exosomes, and exogenous transforming growth factor-β3 (TGF-β3) may further improve exosome production and biological activity [188]. These findings may provide useful guidance for the large-scale production of stem cell-derived exosomes, although culture optimization should be accompanied by rigorous characterization and functional validation. Standardized and practical methods are also needed for exosome isolation and storage. Different isolation or storage methods may affect exosome concentration, purity, morphology, and biological activity, and relevant guidance from the International Society for Extracellular Vesicles should be considered [18]. In addition, the use of serum-free medium may reduce pre-analytical and isolation-related variability. In recent years, combined or sequential isolation techniques have been increasingly applied and may improve exosome purity and yield while reducing the limitations of individual methods [19]. Exosomes can be stored for extended periods at − 80 °C; however, long-term storage and repeated freeze–thaw cycles may affect their biological activity, and higher-temperature or room-temperature storage may alter their physical and biochemical properties [98, 205]. Therefore, unsuitable storage conditions may compromise exosome quality. Freeze-drying with appropriate lyoprotectants, particularly trehalose-sucrose formulations, has been explored as a strategy to preserve the characteristics of stem cell-derived EVs after lyophilization and reconstitution [206, 207].

It is also essential to evaluate the long-term effects of SC-exosomes derived from different stem cell sources across a broad spectrum of renal diseases before they can be developed as clinically applicable therapeutic agents. Safety, quality control, and ethical issues are important considerations for exosome-based therapy. Because exosomes overlap in size with some viral particles, contamination during production or isolation may raise safety concerns [208–210]. Therefore, sterility control, serum-free culture conditions, and enhanced pathogen monitoring are important for SC-exosome preparation. Furthermore, exosome heterogeneity and insufficiently standardized isolation methods may lead to contamination with non-exosomal vesicles, protein aggregates, or other biological components, underscoring the need for standardized isolation protocols and purification techniques. Engineered exosomes have potential advantages, including improved stability, prolonged circulation, targeted delivery, and intracellular cargo release [189], which may help overcome some limitations of natural exosomes. In therapeutic applications, further standardization of SC-exosome dose, administration route, dosing schedule, and potency assessment is also necessary to support the translation of this research into clinical practice.

## Conclusions

In conclusion, kidney diseases remain a major global public health burden, and the development of effective therapeutic strategies continues to be an important focus of renal research. SC-exosomes have emerged as promising cell-free candidates because they can transfer bioactive cargoes and may modulate inflammation, oxidative stress, apoptosis, fibrosis, immune responses, microvascular injury, and tissue repair in experimental renal disease models. Engineered exosomes may further provide opportunities to improve cargo loading, stability, targeting capacity, and therapeutic delivery. However, current evidence remains largely preclinical, and the clinical efficacy and long-term safety of SC-exosome-based therapies have not yet been established. Before routine clinical practice can be considered, further studies are needed to standardize exosome production, isolation, characterization, storage, dosing, delivery strategies, potency assessment, and quality control, and to validate safety and efficacy in well-designed clinical trials.

## Data Availability

No datasets were generated or analysed during the current study.

## Abbreviations

ADSCs: Adipose-derived stem cells
adMSCs: Adipose-derived mesenchymal stem cells
AFM: Atomic force microscopy
AKI: Acute kidney injury
Akt: Protein kinase B
ARE: Antioxidant response element
BMSCs: Bone marrow mesenchymal stem cells
BMSC-exosomes: Bone marrow mesenchymal stem cell-derived exosomes
BUN: Blood urea nitrogen
CKD: Chronic kidney disease
circRNAs: Circular RNAs
DKD: Diabetic kidney disease
DN: Diabetic nephropathy**;**
ECM: Extracellular matrix
EMT: Epithelial–mesenchymal transition
ESCRT: Endosomal sorting complex required for transport
ESKD: End-stage kidney disease
EVs: Extracellular vesicles
GFR: Glomerular filtration rate
hucMSCs: Human umbilical cord mesenchymal stem cells
hucMSC-exosomes: Human umbilical cord mesenchymal stem cell-derived exosomes
hUSCs: Human urine-derived stem cells
hUSC-exosomes: Human urine-derived stem cell-derived exosomes
IgAN: IgA nephropathy
ILVs: Intraluminal vesicles
Keap1: Kelch-like ECH-associated protein 1
LN: Lupus nephritis
lncRNAs: Long non-coding RNAs
miRNAs: MicroRNAs
mRNAs: Messenger RNAs
MSCs: Mesenchymal stem cells
MSC-Exosomes: Mesenchymal stem cell-derived exosomes
mTOR: Mammalian target of rapamycin
MVBs: Multivesicular bodies
NF-κB: Nuclear factor-κB
NLRP3: Nucleotide-binding oligomerization domain-like receptor protein 3
Nrf2: Nuclear factor erythroid 2-related factor 2
NTA: Nanoparticle tracking analysis
PI3K: Phosphatidylinositol 3-kinase
PTEN: Phosphatase and tensin homolog
RCC: Renal cell carcinoma
RIRI: Renal ischemia–reperfusion injury
SC-exosomes: Stem cell-derived exosomes
SEC: Size-exclusion chromatography
SLE: Systemic lupus erythematosus
TGF-β: Transforming growth factor-β
TGF-β1: Transforming growth factor-β1
TLR: Toll-like receptor
TLR4: Toll-like receptor 4
UF: Ultrafiltration
VEGF: Vascular endothelial growth factor
VEGFA: Vascular endothelial growth factor A
YAP: Yes-associated protein
